# Large-scale genome sequencing redefines the genetic footprints of high-altitude adaptation in Tibetans

**DOI:** 10.1186/s13059-023-02912-1

**Published:** 2023-04-13

**Authors:** Wangshan Zheng, Yaoxi He, Yongbo Guo, Tian Yue, Hui Zhang, Jun Li, Bin Zhou, Xuerui Zeng, Liya Li, Bin Wang, Jingxin Cao, Li Chen, Chunxia Li, Hongyan Li, Chaoying Cui, Caijuan Bai, Baimakangzhuo, Xuebin Qi, Ouzhuluobu, Bing Su

**Affiliations:** 1 State Key Laboratory of Genetic Resources and Evolution, Kunming Institute of Zoology, Chinese Academy of Sciences, Kunming, 650223 China; 2 Kunming College of Life Science, University of Chinese Academy of Sciences, Beijing, 100101 China; 3 Fukang Obstetrics, Gynecology and Children Branch Hospital, Tibetan Fukang Hospital, Lhasa, 850000 China; 4 High Altitude Medical Research Center, School of Medicine, Tibetan University, Lhasa, 850000 China; 5 Center for Excellence in Animal Evolution and Genetics, Chinese Academy of Sciences, Kunming, 650223 China

**Keywords:** Tibetan, High-altitude adaptation, Whole-genome sequencing, 1KTGP, Cardio-pulmonary functions, Positive selection, Polygenic effect, Pleiotropic effect

## Abstract

**Background:**

Tibetans are genetically adapted to high-altitude environments. Though many studies have been conducted, the genetic basis of the adaptation remains elusive due to the poor reproducibility for detecting selective signatures in the Tibetan genomes.

**Results:**

Here, we present whole-genome sequencing (WGS) data of 1001 indigenous Tibetans, covering the major populated areas of the Qinghai–Tibetan Plateau in China. We identify 35 million variants, and more than one-third of them are novel variants. Utilizing the large-scale WGS data, we construct a comprehensive map of allele frequency and linkage disequilibrium and provide a population-specific genome reference panel, referred to as 1KTGP. Moreover, with the use of a combined approach, we redefine the signatures of Darwinian-positive selection in the Tibetan genomes, and we characterize a high-confidence list of 4320 variants and 192 genes that have undergone selection in Tibetans. In particular, we discover four new genes, *TMEM132C*, *ATP13A3*, *SANBR*, and *KHDRBS2*, with strong signals of selection, and they may account for the adaptation of cardio-pulmonary functions in Tibetans. Functional annotation and enrichment analysis indicate that the 192 genes with selective signatures are likely involved in multiple organs and physiological systems, suggesting polygenic and pleiotropic effects.

**Conclusions:**

Overall, the large-scale Tibetan WGS data and the identified adaptive variants/genes can serve as a valuable resource for future genetic and medical studies of high-altitude populations.

**Supplementary Information:**

The online version contains supplementary material available at 10.1186/s13059-023-02912-1.

## Background

In recent human evolution, the genetic adaptation of Tibetans to a high-altitude environment is viewed as a classical case [1–3]. Both genetic and archeological data support the Paleolithic settlement (more than 1000 generations) of Tibetans at high altitudes [4, 5], allowing natural selection to enrich genetic mutations conferring adaptation in Tibetans. These known adaptive traits include relatively low hemoglobin levels [6–9], more efficient ventilation, better cardio-pulmonary function [10], and better reproductive viability [11, 12]. Presumably, these traits are shaped by natural selection on multiple genes in the genome.

In the past decade, many studies using genome-wide data have been conducted to search for variants and genes showing signals of Darwinian-positive selection in Tibetans. In total, 682 genes have been reported; however, only two genes (*EPAS1* and *EGNL1*) were successfully replicated in multiple studies, and the reported selective signals of the other genes remain dubious (Additional file 1: Table S1 and S2). Moreover, the published data of genetic association analyses and functional experiments on *EPAS1* and *EGLN1* seem to only account for a small portion of the adaptive traits, including the relatively low hemoglobin level that protects Tibetans from high-altitude polycythemia [10, 13] and better ventilation [14], suggesting that there should be more genes accounting for the other adaptive traits, such as the better cardio-pulmonary function observed in Tibetans. Consequently, the current data still leave many unassigned pieces in the jigsaw puzzle of the genetic adaptation of Tibetans.

The unsolved Tibetan puzzle stems from three major limitations of the current data: (1) small sample size, i.e., all the reported whole-genome-sequencing (WGS) data of Tibetans have fewer than 50 individuals, leading to inaccurate estimation of allele frequencies and limited power in detecting selective signals in the genome; (2) low variant density, i.e., the reported large sample study only generated SNP array data with a limited coverage of the genome [8]; (3) biases in detecting signals of natural selection, i.e., the published studies adopted only one or two methods (but different among studies) to search for selective signals, and the results were inconsistent among studies [1, 6, 8, 15–18]. To overcome these limitations, we need a large-scale WGS data and a combined statistical approach to redefine the footprints of selection in the Tibetan genomes.

In this study, by generating WGS data from 1001 Tibetans, we established a reliable population-specific frequency and linkage disequilibrium (LD) spectrum of Tibetan populations. Furthermore, we systematically evaluated the variants and genes with robust signatures of positive selection, and we provided a high-confidence list of candidate variants (4320 variants) and genes (192 genes) potentially underlying the genetic basis of Tibetans’ adaptation to high altitude. Notably, 152 genes (79%) are newly identified genes. In particular, we discovered five genes with strong selective signals that may explain the better lung function of Tibetans.

## Results

### Whole-genome sequencing of 1001 Tibetans

**Fig. 1:**
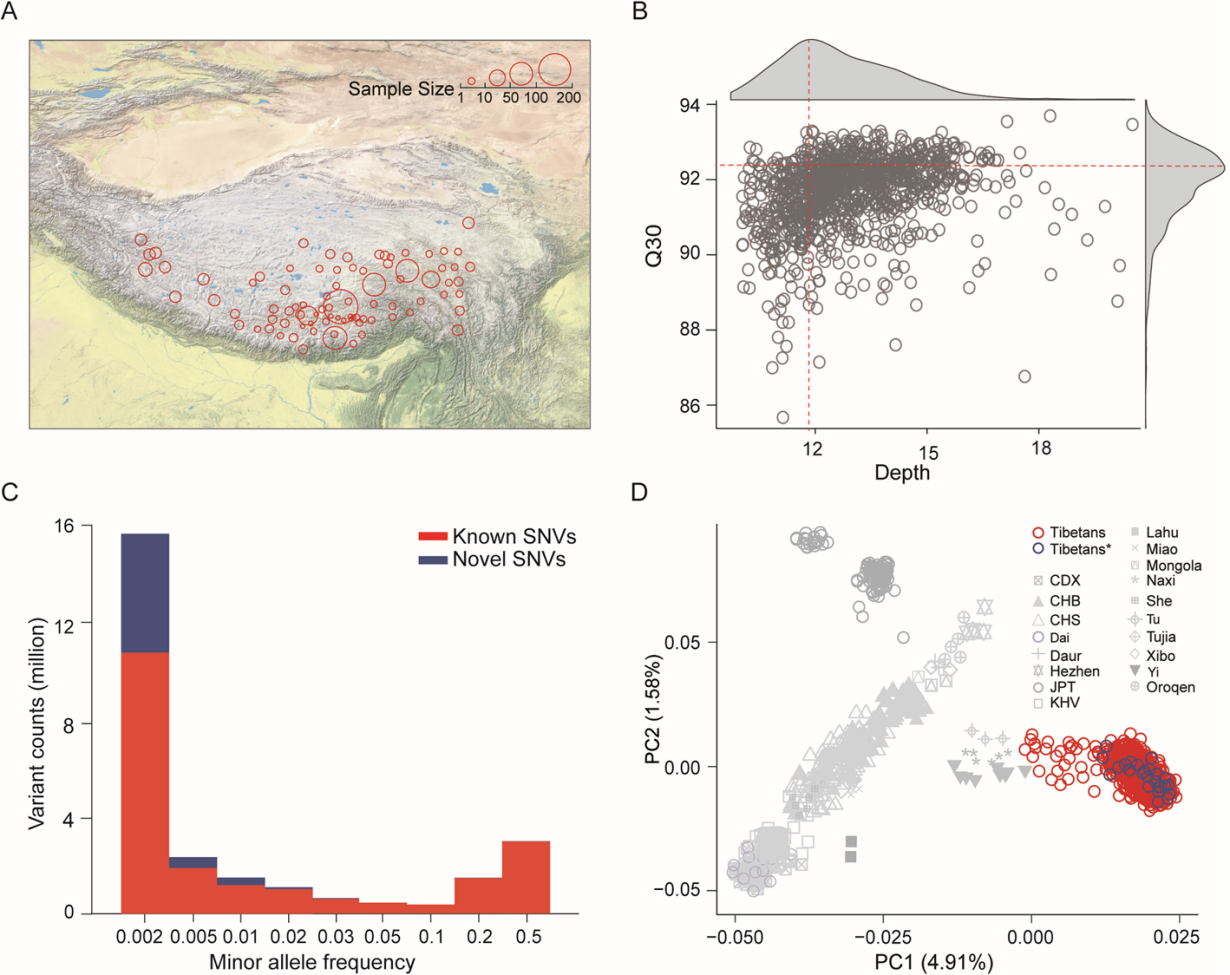
Geographic locations of the sampled Tibetans and WGS data quality assessment. **A** The geographical locations of the Tibetan samples in this study. The sampling locations and the sample sizes are indicated. **B** The quality of the Tibetan 1001 WGS data, reflected by the depth and Q30 values. The mean depth and Q30 are indicated with the red dotted lines. **C** The minor allele frequency spectrum of all identified SNVs. The known and novel variants are shown in red and blue, respectively. **D** The genome-wide PCA plot of Tibetans and 18 representative East Asian populations. The red circles are the 1001 samples (Tibetans) from the current study, and the blue circles are the 33 published WGS samples (Tibetans*) [18]

We recruited 1064 Tibetan participants from 83 different geographic locations (altitude range: 2300–4900 m) of the Qinghai–Tibetan Plateau in China (Fig. 1A, Additional file 1: Table S3). WGS was performed for these individuals with a mean depth of 11.8 × and data quality mean Q30 of 93% (Fig. 1B). After stringent filtering (see the “Methods” section), we kept the WGS data of 1001 individuals for genome-wide variant calling and downstream analyses. Using the standard GATK pipeline (see the “Methods” section), we identified 34.7 million variants among the 1001 Tibetans, including 29.9 million single-nucleotide variants (SNVs) and 4.8 million INDELs (insertions and deletions with size < 50 bp). Among the 28.2 million biallelic SNVs, 36% are novel SNVs not reported in the dbSNP database (build 154) (Fig. 1C, Additional file 1: Table S4).

We then conducted principal component analysis (PCA) by including the 1001 Tibetans and 6527 global individuals from 1000 Genome Project-Phase III (referred to as 1KGP3) and Human Genome Diversity Project (HGDP), as well as the 33 published Tibetans [18]. In the PCA map covering global populations, Tibetans show a close relationship with other East Asian populations (Additional file 2: Fig. S1A). The genetic relatedness between Tibetans and the other populations is consistent with the results from the reported array data [8, 9, 11]. When only East Asian populations were included, the 1001 Tibetans cluster tightly with the 33 Tibetans, and together they formed a separate group from the other East Asian populations (Fig. 1D). Of note, the three ethnic groups from China (Tu, Naxi, and Yi) are relatively close to Tibetans (Fig. 1D), and all of them live in the surrounding areas of the Qinghai–Tibetan Plateau with known admixture with Tibetans. The genome-wide heterozygosity ratio of Tibetans is 1.41 ± 0.046, and the divergence between Tibetans and Han Chinese (*F*_ST(Tibetan-Han)_) is 0.0095 (Additional file 2: Fig. S1), similar to the reported array data [8] and the WGS data with a small sample size [18]. Collectively, with a large sample size and a wide geographic coverage, the large-scale WGS data of Tibetans should be highly informative in understanding the genetic structure of indigenous Tibetan populations.

By conducting functional annotation, we characterized 173,345 high-impact variants in the 1001 Tibetan genomes, including 8279 loss-of-function variants and 164,616 missense variants. Notably, we identified 391 novel missense mutations that are relatively common (> 3%) in Tibetan populations (Additional file 2: Fig. S1 and Additional file 1: Table S5).

Genomic structural variations (SVs) may play a key role in human genetic adaptation and disease susceptibility [19, 20]. We previously de novo assembled a Tibetan genome using long-read sequencing data and generated a high-confidence SV set (approximately 17,900 SVs) with precise breakpoints [21]. Here, we sought to estimate the population frequencies of these SVs in Tibetan populations by leveraging our large-scale WGS data. To this end, we set up a pipeline for genotyping and filtering SVs in Tibetan populations using the WGS short-read data based on the well-solved breakpoint set (see the “Methods” section). We successfully genotyped 9490 SVs, including 3135 deletions, 34 duplications, 6290 insertions, and 31 inversions (Additional file 2: Fig. S2A, S2B). Among the 9490 SVs, 1523 (16.05%) SVs are low-frequency variants (< 1%), and the functional annotation showed that half of the SVs (54.3%) were located in the noncoding regions, including 1963 SVs (20.6%) in the annotated regulatory regions with potential functions (Additional file 2: Fig. S2C, S2D).

### The spectrum of variant frequency and linkage disequilibrium in Tibetans

**Fig. 2:**
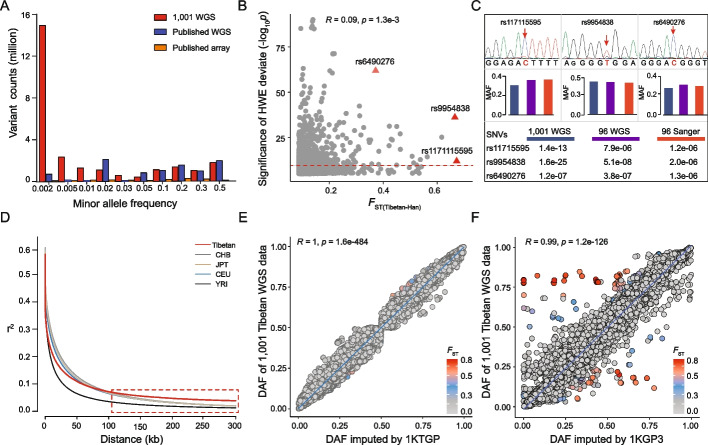
The spectrum of genome-wide variant frequency and LD of Tibetans. **A** Comparison of SNV counts of MAF among the 1001 WGS data and the published data. The 1001 WGS data is much more powerful in detecting rare variants than the published data. **B** The distribution of HWE deviation for SNVs with large between-population divergences (*F*_ST(Tibetan-Han)_ > 0.1), and the cutoff of HWE deviation is 1e − 6. **C** Validation by Sanger sequencing of three HWE-deviated SNVs with high *F*_ST(Tibetan-Han)_. The top panel shows the electro-morph of Sanger sequencing of the three SNVs. The histograms in the middle indicate the minor allele frequencies (MAF) of the three SNVs from three datasets, including the WGS data of 1001 Tibetans (in blue), the 96 random samples from the 1001 WGS data (in green), and the Sanger sequencing data of 96 samples (in red). The *p* values under the histograms indicate the significance levels of HWE deviation of the three SNPs based on the three datasets.** D** Comparison of the LD decay patterns between Tibetans and other world populations. The dashed box indicates a distinctive LD decay pattern of Tibetans. For the decay of long genomic regions (> 100 kb), Tibetans show a slower decay (reflected by the higher *r*.^2^ values) than those of other world populations, an indication of extended haplotype homozygosity. **E** The correlation of DAF (derived allele frequency) of the genome-wide SNVs from the 1001 Tibetan WGS data and the 3008 Tibetan array data [8] imputed by 1KTGP. **F** The correlation map when imputed by 1KGP3

Large-scale WGS data is ideal to construct a refined genomic architecture of a population. Here, we established the spectrum of genome-wide variant frequencies and LD of Tibetans based on the 1001 genome sequences. Compared to the previous studies of Tibetan WGS with small sample sizes [15, 16, 18] or with a large sample size of array data [8], our variant set exhibited remarkable power for detecting rare variants (minor allele frequency, MAF < 3%) and unbiased coverage of the entire genome (Fig. 2A).

Based on the established frequency spectrum, we tested a key index of population structure: Hardy–Weinberg equilibrium (HWE). In most genomic studies, especially genome-wide association studies (GWAS), the variants that show deviation from HWE are usually filtered out because they are indicative of genotyping or genotype-calling errors [8, 11, 15, 22]. The commonly used significance threshold for HWE deviation varies from 1e − 3 to 1e − 6 in the literature [22]. However, it is known that deviations from HWE may also indicate natural selection other than genotype errors, and previous studies have not evaluated this point effectively in a proper population. Given that Tibetans have been subject to a strong natural selection of extreme environments, they serve as an ideal population for this purpose. We performed the HWE deviation test for genome-wide variants in Tibetans. We noticed that there were 207 variants showing significant HWE deviations (*p* < 1e − 6), and at the same time, they also showed deep divergences between Tibetans and Han Chinese, an indication of strong natural selection (*F*_ST(Tibetan-Han)_ > 0.2) (Fig. 2B). These variants are all in robust quality and passed all rigorous QCs, including read depth, mapping quality (MQ), base quality (BQ), and genotype quality (GQ) (Additional file 2: Fig. S3A). To further validate these HWE-deviated variants and to exclude the possibility of sequencing errors, we selected three variants (rs117115595, rs9954838, and rs6490276) with high *F*_ST(Tibetan-Han)_ values (Fig. 2B) and conducted Sanger sequencing among 96 randomly selected Tibetans. The genotype frequencies and the HWE patterns of the three selected variants are highly consistent with the WGS data (Fig. 2C), suggesting that the HWE-deviated variants are most likely true mutations undergone positive selection in Tibetans, rather than genotyping errors. Our HWE analysis demonstrates that the significance threshold of HWE deviation should be looser for populations (such as Tibetans) undergoing strong natural selection.

Linkage information is crucial for inferring common variants by imputation when dealing with array data, and the selection of reference WGS panels can greatly affect the accuracy of imputation [23–27]. Due to strong natural selection, the LD decay pattern of Tibetans is highly distinctive from other world populations, especially for the genomic regions with extended haplotype homozygosity (> 100 kb) (Fig. 2D). Here, we reconstructed a Tibetan-specific reference panel of LD using our 1001 genome sequences (referred as 1KTGP), and we evaluated the imputation accuracy of the published array data that used the reference panel of global populations (referred as 1KGP3) [8]. It turned out that the 1KTGP-imputed genotype frequencies are highly consistent with the 1001 WGS data (Fig. 2E). In contrast, the 1KGP3-imputed genotype frequencies have many SNVs with serious deviations from the 1001 WGS data (Fig. 2F), e.g., the SNVs located in the *EPAS1* gene region (Additional file 2: Fig. S3B, S3C). The results suggest that our Tibetan LD panel is much better for the imputation of Tibetan array data, and this high-resolution and population-specific reference panel serves as a valuable resource for future Tibetan population studies.

### Redefine the genomic signatures of positive selection in Tibetans

**Fig. 3:**
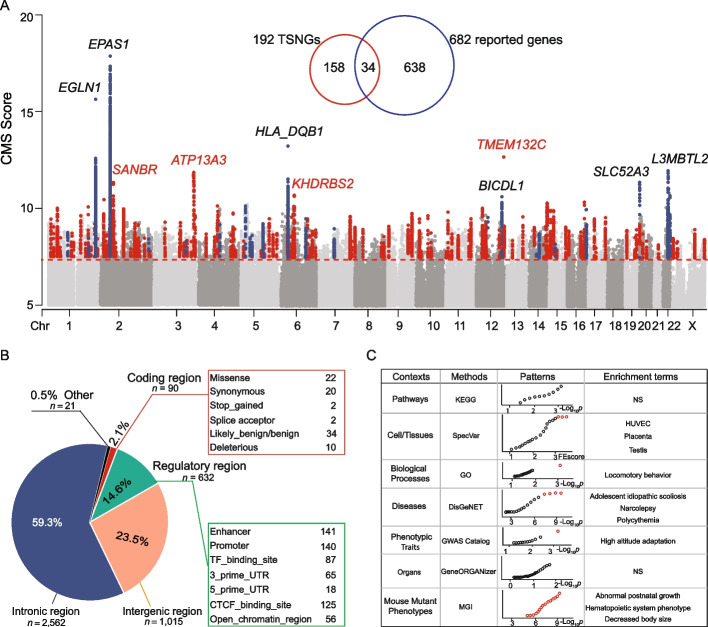
Genome-wide signals of Darwinian-positive selection in Tibetans.** A** The distribution of the CMS scores of the genome-wide SNVs in Tibetans. The 192 lead gene regions are marked in red (newly identified genes) and blue dots (reported genes). The top 10 TSNGs are indicated with gene names (4 newly identified and 6 reported). The Venn plot shows the overlap between the reported gene set and the identified gene set in this study. **B** Functional annotations of the 4320 TSNSs. The “Regulatory region” refers to the noncoding region with regulatory annotations. **C** The functional enrichment patterns of TSNGs using different methods. The significant terms are indicated in red in the bubble plots. NS, not significant

To detect the genomic variants contributing to the adaptation of Tibetans, with the use of the 1001 WGS data, we applied the method considering the composite of multiple signals (CMS) [28] to identify variants under positive selection. We first generated the variant set with the top 1‰ CMS scores (> 7.66) and then filtered the set by only keeping the variants showing Tibetan-specific enrichment, i.e., the Tibetan enriched-allele frequency of the variant is higher in Tibetans when compared to four major global populations (Han Chinese, Japanese, Europeans, and Africans) (see the “Methods” section). The final set contains 4320 variants, which were taken as the Tibetan selection-nominated SNVs (TSNSs). They are located in 236 independent genomic regions, represented by 192 lead genes, referred to as the Tibetan selection-nominated genes (TSNGs). Of the 192 TSNGs, 34 were reported previously, and the other 158 are newly identified genes (Fig. 3A, Additional file 2: Fig. S4 and Additional file 1: Table S6).

We conducted functional annotation for the 4320 TSNSs. The majority of them (75.4%) are located in the noncoding regions, and 632 of them (14.6%) fall in the annotated regulatory regions. There are 90 TSNSs in the coding regions, including 22 missenses, 20 synonymous, 2 stop-gained, 2 splice acceptor, 10 deleterious, and 34 likely_benig/benign (Fig. 3B; Additional file 1: Table S7). Among the 22 missense TSNSs, 13 are highly enriched in Tibetans showing > 20% higher frequencies in Tibetans compared to other global populations, and four of them were previously reported, including *EGLN1* (rs186996510), *TMEM247* (rs116983452 and rs12612916), *ADH1B* (rs1229984), and *OCA2* (rs1800414), while the other ten are newly identified in this study (Table 1). For example, rs79703522 is a missense mutation of *RP11-766F14.2* (encoding a functionally unknown protein expressed in the muscle, heart, and kidney), which is predominant in Tibetans (87%), and on average 59% higher than in other global populations (Table 1). Notably, there were 54 reported missense and loss-of-function mutations with enriched frequencies in Tibetans in the previous study [15], and we found only 5 of them could be successfully validated in our large-scale WGS data (with *F*_ST(Tibetans-Han)_ > 0.1), suggesting that a large sample size is vital for accurately estimating the allele frequencies (Table 1, Additional file 1: Table S7).

**Table 1:** The 14 highly enriched missense variants in Tibetans

CHR	POS (v.37)	SNVs	Gene	Genotype	Amino acid	CMS score	*F*_ST_	Derived allele frequency				
								**TBN**	**CHB**	**JPT**	**CEU**	**YRI**
1	231557623	rs186996510	*EGLN1*	c.12C > G	p.Asp4Glu	15.63	0.53	0.7	0.03	0.06	0	0
2	46707674	rs116983452	*TMEM247*	c.248C > T	p.Ala83Val	14.16	0.54	0.7	0.02	0.04	0	0
2	46707886	rs12612916	*TMEM247*	c.460G > A	p.Glu154Lys	12.22	0.32	0.85	0.51	0.54	0.04	0.12
**1**	**231488524**	**rs2437150**	***SPRTN***	**c.887C > T**	**p.Pro296Leu**	**11.66**	**0.23**	**0.78**	**0.46**	**0.48**	**0.39**	**0.56**
15	28197037	rs1800414	*OCA2*	c.1844A > G	p.His615Arg	9.76	0.51	0.11	0.59	0.59	0.12	0.11
**1**	**42898843**	**rs1034268**	***ZMYND12***	**c.946 T > C**	**p.Phe316Leu**	**8.90**	**0.17**	**0.66**	**0.36**	**0.48**	**0.35**	**0.46**
**2**	**46706765**	**rs192690066**	***TMEM247***	**c.41C > T**	**p.Ala14Val**	**8.85**	**0.16**	**0.26**	**0**	**0**	**0**	**0**
**4**	**100574821**	**rs79703522**	***RP11-766F14.2***	**c.985G > C**	**p.Gly329Arg**	**8.41**	**0.27**	**0.87**	**0.44**	**0.42**	**0.14**	**0.12**
**7**	**38284804**	**rs4629747**	***TRGC2***	**c.356A > G**	**p.Asn119Ser**	**8.37**	**0.2**	**0.39**	**0.06**	**0.06**	**0.07**	**0.02**
4	100239319	rs1229984	*ADH1B*	c.143A > G	p.His48Arg	8.11	0.56	0.86	0.29	0.27	0.02	0
**2**	**61385105**	**rs1729671**	***SANBR***	**c.93G > C**	**p.Trp31Cys**	**8.03**	**0.21**	**0.92**	**0.7**	**0.65**	**0.61**	**0.58**
**2**	**61385100**	**rs1665258**	***SANBR***	**c.88G > A**	**p.Glu30Lys**	**7.72**	**0.21**	**0.92**	**0.7**	**0.65**	**0.61**	**0.58**
**12**	**113376388**	**rs1859330**	***OAS3***	**c.53G > A**	**p.Arg18Lys**	**7.69**	**0.22**	**0.92**	**0.7**	**0.84**	**0.62**	**0.54**
**19**	**8000104**	**rs3745387**	***TIMM44***	**c.347 T > C**	**p.Val116Ala**	**7.67**	**0.13**	**0.73**	**0.48**	**0.49**	**0.44**	**0.69**

The missense TSNSs in bold are those newly identified in this study

*CHR* Chromosome, *POS (v.37)* Physical position of GRCh37

For the 192 TSNGs, we performed functional enrichment analyses using various methods to reveal the involved pathways, cell types/tissues, biological processes, related diseases, phenotypic traits, organ systems, and mouse-knockout phenotypes. In general, the enriched functional terms nicely reflected varied adaptive physiological traits in Tibetans. For instance, for cell types and tissues, these TSNGs were significantly enriched in human umbilical vein endothelial cells (HUVECs), placenta, and testis, reflecting the adaptive changes of Tibetans in oxygen sensing (endothelial cells), development (placenta), and reproductive viability (testis), which are consistent with the mouse-knockout phenotypes (abnormal postnatal growth and decreased body size), as well as the disease term (adolescent idiopathic scoliosis) (Fig. 3C, Additional file 1: Tables S8-S13). Better oxygen transportation in Tibetans was clearly shown in the disease catalog of polycythemia and the mouse-knockout phenotype of the hematopoietic system (Additional file 1: Table S11 and S13). Interestingly, we also detected two functional terms involving the brain (locomotory behavior and narcolepsy) (Additional file 1: Table S10 and S11). Since the brain is the most oxygen-consuming organ, we speculate that high-altitude hypoxia might have also selected some advantageous mutations in genes helping maintain the normal function of the central nervous system.

In addition, we identified 76 SVs with large frequency divergences (> 20%) between Tibetans and other global populations, referred to as the Tibetan-enriched SVs (TESVs) (Additional file 1: Table S14, Additional file 2: Fig. S5). The top TESV is the well-known 3.4-kb deletion in *TMEM247* (also known as TED) with Tibetan-specific enrichment (70.7% in Tibetans vs 7% in Han Chinese) [29]. Consistent with recent studies using long-read sequencing data, we successfully replicated the three reported TESVs, including the 163-bp deletion in *MKL1* [21], the 2590-bp deletion in *ZFAND2A*, and the 194-bp deletion in *SMAD2* [30]. We also found 71 novel TESVs involving 43 genes, including 27 deletions, 16 insertions, and 1 duplication (Additional file 2: Fig. S5, Additional file 1: Table S14). For example, the newly identified 322-bp intronic deletion in *PKHD1L1* (PKHD1 ciliary IPT domain containing fibrocystin/polyductin like 1) is enriched (49.5%) in Tibetan populations, but relatively rare (< 23%) in non-Tibetan populations. This gene is reportedly associated with sleep quality, a known physiological response at high altitudes [31–33], echoing the aforementioned term of narcolepsy in the functional enrichment analysis of the 192 TSNGs (Fig. 3C).

Recent studies have suggested that archaic hominin introgression may play an important role in facilitating biological adaptation to new environments. For example, the adaptive *EPAS1* haplotype was proven as a “borrowed fitness” from Denisovans [34]. To search for the genome-wide regions showing signatures of archaic introgression in the Tibetan genomes, we employed two widely used tools, ArchaicSeeker2.0 [35] and SPrime [36] (see the “Methods” section), which gave 599 and 670 candidate introgression regions, respectively. The detected genome-wide introgression levels (0.06–0.18% from Denisovan and 0.34–0.79% from Neanderthal) of Tibetans are similar to those reported in other East Asian populations [35]. There are only 25 shared regions between the sets generated by the two different tools, which we take as the high-confident introgression regions (Additional file 1: Table S15). Five of the 25 regions are overlapped with the identified gene regions under selection in the Tibetan genomes, including *EPAS1*, *EGLN1*, *DISC1*, *PRKCE*, and *FANCA*, and they were also reported in the previous study [37] (Additional file 1: Table S15; Additional file 2: Fig. S6). It should be noted that the detection of introgression usually requires deep sequencing data (> 30 ×) for high-quality phasing. Considering the relatively low sequencing depth (~ 11.8 ×) of our data, future deep sequencing, especially the long-read data, should be informative to further test the introgression pattern in Tibetans.

### The newly identified top TSNGs explain the adaption of cardiopulmonary function in Tibetans

**Fig. 4:**
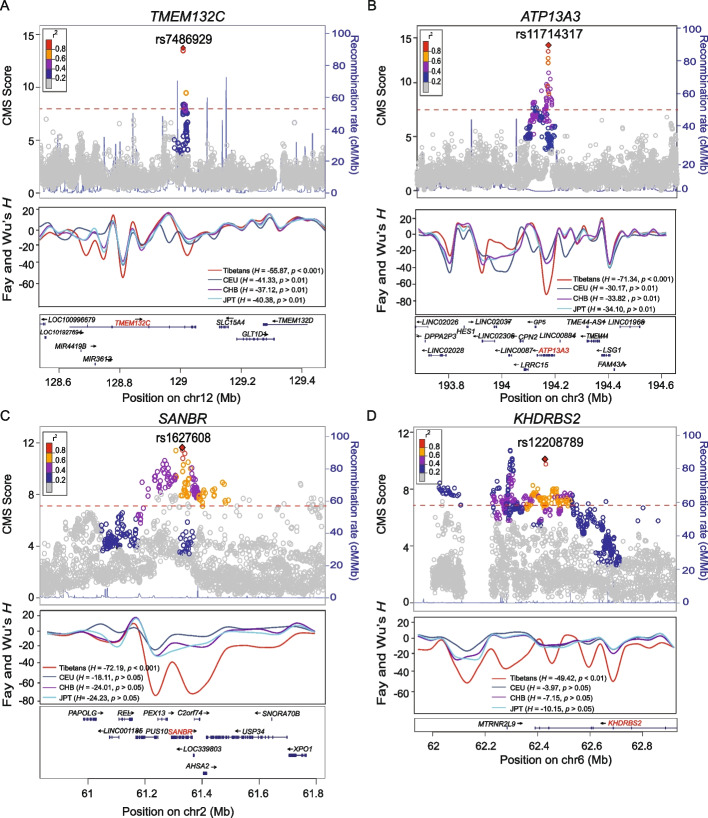
Four newly identified TSNGs in the top 10 signals. **A**–**D** The regional plots of CMS scores and recombination rates, in which the peaks indicate the selective signals. The peak SNVs are marked with colors. The results of sliding window Fay and Wu’s *H* tests of the four genes are also presented. **A** The *TMEM132C* gene region. **B** The *ATP13A3* gene region. **C** The *SANBR* gene region. **D** The *KHDRBS2* gene region. The calculated recombination rates (*r*^2^) indicate the estimated linkage disequilibrium (LD) degree between the peak SNV and the other SNVs and are coded in colors. The significance threshold of CMS = 7.66 (top 1‰) is denoted by the red dashed line. The *H* values refer to the maximum scores of the given regions (marked in red), covering the upstream and downstream 500-kb regions of the peak SNVs of the four genes

Among the top 10 TSNGs, in addition to the six previously reported genes (*EPAS1*, *EGLN1*, *HLA_DQB1*, *L3MBTL2*, *SLC52A3*, and *BICDL1*) [6, 8, 9, 17, 18, 38], we identified four new genes with strong selection, including *TMEM132C*, *ATP13A3*, *SANBR*, and *KHDRBS2* (Fig. 4, Additional file 2: Fig. S7 and Table 2). It is known that better cardiopulmonary function represents the key adaptation of Tibetans [39–41]; however, the genes responsible for this adaptation have not been discovered. Remarkably, the four newly identified top TSNGs provided clues to this unsolved question.

**Table 2:** The top 10 selection signals in the Tibetan genome

CHR	POS (v.37)	SNVs	Genotype	TEA	Gene	CMS score	*F*_ST_	Frequency of TEA				
								**TBN**	**CHB**	**JPT**	**CEU**	**YRI**
2	46,589,032	rs74898705	C > T	T	*EPAS1*	17.86	0.68	0.80	0.01	0.00	0.03	0.16
1	231,557,623	rs186996510	G > C	C	*EGLN1*	15.63	0.53	0.70	0.03	0.05	0.00	0.00
6	32,629,955	rs1063321	C > T	T	*HLA_DQB1*	13.26	0.27	0.82	0.46	0.52	0.39	0.29
**12**	**129,084,774**	**rs7486929**	**A > G**	**A**	***TMEM132C***	**12.71**	**0.29**	**0.90**	**0.60**	**0.56**	**0.52**	**0.57**
**3**	**194,151,467**	**rs79377418**	**C > A**	**A**	***ATP13A3***	**11.72**	**0.13**	**0.32**	**0.00**	**0.02**	**0.11**	**0.10**
22	41,627,775	rs5751069	G > C	C	*L3MBTL2*	11.6	0.29	0.91	0.61	0.78	0.66	0.45
**2**	**61,330,217**	**rs1729672**	**A > G**	**A**	***SANBR***	**11.33**	**0.30**	**0.94**	**0.68**	**0.71**	**0.72**	**0.88**
20	753,310	rs6117562	A > G	G	*SLC52A3*	11.14	0.47	0.97	0.64	0.58	0.77	0.67
**6**	**62,431,522**	**rs12208789**	**A > G**	**A**	***KHDRBS2***	**10.7**	**0.42**	**0.91**	**0.51**	**0.50**	**0.57**	**0.81**
12	120,434,838	rs11064986	C > T	T	*BICDL1*	10.7	0.27	0.83	0.47	0.46	0.81	0.47

The four newly identified TSNGs are highlighted in bold

*TEA* Tibetan-enriched allele, *CHR* Chromosome, *POS (v.37)* Physical position of GRCh37

*TMEM132C* (transmembrane protein 132C) is a member of the *TMEM132* family, and its molecular function remains unclear. The peak SNV rs7486929 (CMS = 12.71) is located in the intronic region of *TMEM132C* with Tibetan-specific enrichment (ΔDAF > 29% between Tibetans and other global populations). The selection signal of *TMEM132C* is further confirmed by Fay and Wu’s *H* test (*H* =  − 55.87, *p* < 0.001) (Fig. 4A; Table 2). The previous family-based study reported that mutations in this gene were associated with pulmonary and lung function (forced expiratory volume in 1 s (FEV1)) [42].

*ATP13A3* (ATPase 13A3) is a member of the P-type ATPase family that transports a variety of cations across cell membranes, and they serve as the major component of the mammalian polyamine transport system. The top SNV rs11714317 showed a strong selection signature with a distinctive LD decay pattern in Tibetans compared to other populations (CMS = 11.7, XPEHH = 5.7). The selection of *ATP13A3* is further validated by Fay and Wu’s *H* test (*H* =  − 71.34, *p* < 0.001) (Fig. 4B; Additional file 1: Table S6). Additionally, we identified multiple TSNSs with annotated regulatory roles. For example, rs71316300 (CMS = 8.4) is located in an enhancer, and rs75122941 (CMS = 8.2) is located in the promoter of *ATP13A3*. *ATP13A3* plays important roles in lung vascular remodeling and pulmonary arterial hypertension (PAH) [43–45]. Hence, the Tibetan-enriched mutations of *ATP13A3* may protect Tibetans from pulmonary hypertension (PAH).

*SANBR* (SANT and BTB domain regulator of CSR, also known as *KIAA1841*) is known for its association with peroxisome biogenesis disorder. The selective signals of *SANBR* are consistent among various statistics, including Fay and Wu’s *H* test (*H* =  − 72.19, *p* < 0.001). The top SNV rs1627608 is highly diverged between Tibetans and Han Chinese (*F*_ST(Tibetan-Han)_ = 0.25), and based on the GTEx database, it is an expression quantitative trait locus (eQTL) in the testis, lung, artery-aorta, muscle-skeletal, and heart-atrial appendage (Additional file 2: Fig. S8). Furthermore, we also identified two missense *SANBR* variants under positive selection (*F*_ST(Tibetan-Han)_ > 0.2) (Table 1).

*KHDRBS2* (KHRNA-binding domain containing, signal transduction associated 2) is a RNA-binding protein acting on the regulation of alternative splicing, and it is abundantly expressed in the lung and brain [46]. The top SNV rs12208789 is located in the intronic region of *KHDRBS2* and shows a strong positive selection in Tibetans with > 32% higher frequency in Tibetans than in other global populations (Fig. 4D; Table 2). Large-population GWAS studies showed that *KHDRBS2* was associated with lung function (FEV/FEC ratio) and atrial septal defects [47, 48].

Collectively, the functions of the four newly identified genes are closely related to the cardiopulmonary system, suggesting that multiple genes harbor enriched adaptive mutations in Tibetans, and they may work together to improve the cardiopulmonary function under hypobaric hypoxia. Future functional experiments on these genes are warranted to reveal the underlying regulatory mechanisms and phenotypic outcomes.

### Polygenic and pleiotropic effects of genetic adaption in Tibetans

**Fig. 5:**
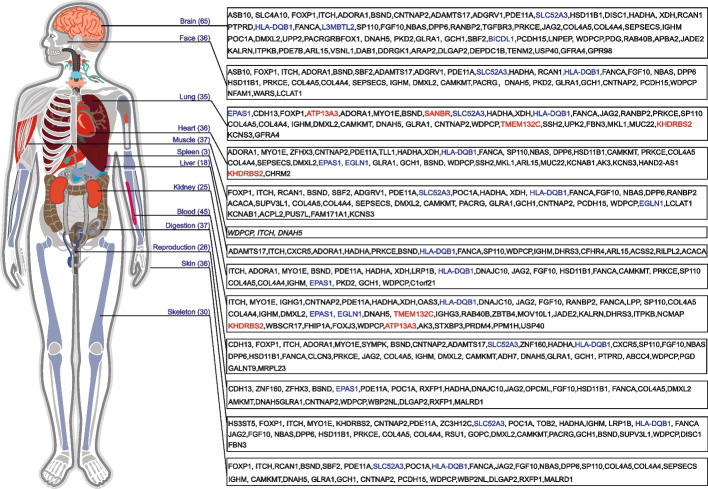
The polygenic and pleiotropic effects of the 192 TSNGs. The genes are assigned to different organs or physiological systems based on the current functional databases by using GeneORGANizer [51]. The top 10 TSNGs are highlighted by bold font in red (newly identified) and in blue (reported)

To determine how the 192 identified TSNGs contribute to the adaptation of various physiological systems in Tibetans, we conducted the GeneORGANizer analysis so that the genes could be assigned to different organs/systems of the body based on their functional annotations. It turned out that these TSNGs function in multiple organs/systems, including those with known adaptive features in Tibetans, such as the blood (45 genes), lung (35 genes), heart (36 genes), and reproduction (26 genes), as well as those without known connections to adaptation, such as the brain (65 genes), face (36 genes), muscle (37 genes), kidney (25 genes), digestion (37 genes), skin (36 genes), and skeleton (30 genes) (Fig. 5 and Additional file 1: Table S8). Markedly, many TSNGs seem to work in multiple organs/systems, an implication of pleiotropic effects of these genes in regulating the physiological adaptation of Tibetans. For example, *HLA-DQB1* is among the top 10 TSNGs, and it functions in almost all the listed organs/systems (Fig. 5) due to its role in the immune system [49]. Similarly, *SLC52A3* appears in 7 different organs/systems, implying that besides the known role of *SLC52A3* in the brain [50], it may also contribute to the adaptation of other organs.

There are 65 TSNGs (34%) in the brain, including 4 of the top 10 TSNGs (*SLC52A3*, *HLA-DQB1*, *L3MBTL2*, and *BICDR1*). These four top genes have known functions in the brain, including AD pathology [50], neuroticism [52], and regulation of neurite outgrowth. Hence, as the most oxygen-consuming organ, the brain should be one of the target organs of natural selection at high altitudes, though it was largely ignored in previous studies except for the studies on sleep [10]. It would be worthwhile to compare the cognitive performance and brain disease susceptibility between Tibetans and the lowlanders living at high altitudes.

It is reported that hypoxia can generate harmful effects on the maintenance of intestinal homeostasis [53]. There are 30 TSNGs in the digestive system. As we mentioned above, *SCL52A3* not only works in the brain, but also in other organs/systems including the digestive system. *SCL52A3* encodes a riboflavin transporter protein with abundant expression in the intestine, and it plays a role in the intestinal absorption of riboflavin [54]. Therefore, one of the adaptive roles of *SCL52A3* might be maintaining the intestinal homeostasis of Tibetans under hypoxic conditions.

Another important organ is the skin, which contains 35 TSNGs. At high altitudes, besides hypobaric hypoxia, UV radiation is another environmental condition that can cause strong natural selection because the intensity of UV radiation at the Qinghai–Tibetan Plateau is comparable to the near-equator regions such as mainland Southeast Asia [55]. It was reported that Tibetans have darker skin compared to lowland Han Chinese, an implication of adaptive skin color change to cope with the strong UV radiation at altitude. Among the 35 skin-related TSNGs, 6 of them are indeed involved in the pigmentation pathway [49].

The 192 identified TSNGs show both polygenic and pleiotropic effects on the genetic adaptation of Tibetans to high altitude, and they may work in multiple organs and physiological systems to improve the viability and reproductive success of Tibetans (Fig. 5). The functional roles, the regulatory mechanisms, and molecular interactions of these TSNGs call for future in-depth studies.

To further confirm the proposed polygenic adaptation of Tibetans, we also conducted an SNV-based analysis of polygenic selection. The GWAS data from the UK Biobank of the blood index (blood hemoglobin level (HGB)) was used, including 2031 independent HGB-associated SNVs. The PolyGraph tool was employed in the analysis [56] (see the “Methods” section for details). The results show that Tibetans have a clear signature of polygenic selection on the HGB level (*p*_adjust_ = 1.38e − 20), and the pattern remains the same when excluding the *EPAS1* SNVs (*p*_adjust_ = 1.13e − 12) (Additional file 2: Fig. S9).

## Discussion

The genetic adaptation of Tibetans likely involves a group of genes harboring advantageous mutations [5–9, 15–18, 38, 57, 58]. In this study, we generated a large-scale WGS data from 1001 Tibetans, providing an unbiased coverage of genome-wide variants, from which we constructed a refined frequency and LD spectrum. We highlighted the value of the genome reference panel containing high-quality genome sequences of Tibetan populations (the 1KTGP panel). With the use of the combined statistics, we systematically redefine the footprints of selection in the Tibetan genome, and identified a confident set of 192 genes showing signatures of positive selection in Tibetans, and 158 of them are newly reported genes in this study.

We reckon that among the 192 TSNGs, only 34 of them were reported in previous studies. In other words, the great majority of the previously reported 682 genes could not be verified in our large-scale WGS data. For example, *MTHFR* and *PPARA* are the two well-known genes with previous evidence of selection [8, 17]. However, based on our data, the previous signals of these two genes were probably false positive because neither of them showed evidence of selection (CMS = 4.6, *F*_ST(Tibetan-Han)_ = 0.023, iHS < 2, XPEHH < 2 for *MTHFR*; CMS = 4.93, *F*_ST(Tibetan-Han)_ = 0.07, iHS < 2, XPEHH < 2 for *PPARA*) (Additional file 2: Fig. S10A, S10B), likely due to the aforementioned drawbacks of the published data.

A sufficiently large sample size and high-coverage variants are crucial for resolving the key questions in population genetics and medical genetics [2, 59]. Although many genome-wide Tibetan studies have been conducted, most of the proposed positively selected genes could not be validated by independent studies due to the limitation of the previous data. One of the statistical issues is allele frequency fluctuations caused by small samples (Additional file 1: Table S1). Our 1001 WGS data overcame this problem and provided credible variants and genes under strong selection in Tibetans (Fig. 3A, Additional file 1: Table S6). Another issue is the limited coverage of variants in the published array-based data, and we fixed this problem by generating high-density genomic variants. Compared to the previous array data, WGS data generated an unbiased and high-coverage SNV distribution of the entire genome, providing a valuable Tibetan reference panel of genetic variants. Previously, there was a lack of a proper reference panel to perform imputation for low-density array data. The commonly used human population reference panels (1KGP3) may bring bias for imputing genotypes of underrepresented populations (such as Tibetans), leading to distortion of population-specific genetic architecture [25, 60, 61] (Fig. 2E). We demonstrated that the Tibetan reference panel (1KTGP) had a superior performance in view of an imputed number of variants and imputation accuracy compared to the 1KGP3 panel, especially for those variants located in the genomic regions undergone strong selection in Tibetans (Fig. 2D, Additional file 2: Fig. S3B, S3C).

A single method for detecting natural selection usually brings false-positive or false-negative signals. In previous studies, there were only a few using more than two different methods (frequency-based or haplotype-based methods) to detect selection [15, 16]. Consequently, many of the reported 682 genes under selection in Tibetans were based on a single method with poor reproducibility. For example, *VDR* and *DNMBP* were identified by frequency-based and haplotype-based methods, respectively. In our study, we found that *VDR* showed a selection signal only in the frequency-based method (*F*_ST(Tibetan-Han)_ = 0.15) but not in the haplotype-based method (iHS < 2, XPEHH < 2) (Additional file 2: Fig. S11A). Similarly, *DNMBP* showed a selection signal only in the haplotype-based method (iHS = 4.7, XPEHH = 1.3) but not in the frequency-based method (*F*_ST(Tibetan-Han)_ < 0.1). Thus, these genes are likely false positive, and they were not identified in our list of 192 TSNGs based on the combined statistics incorporating both frequency-based (*F*_ST(Tibetan-Han)_ and ΔDAF) and haplotype-based (iHS and XPEHH) methods (Additional file 2: Fig. S11B).

It should be noted that the 192 identified TSNGs likely represent a conservative set of genes because the applied cutoffs (top 1‰ CMS and Tibetan-specific enrichment alleles) are relatively stringent to identify genes under strong selection in Tibetans. Therefore, we might miss genes under weak selection. Additionally, because we defined a TSNG falling in the top signal spanning an independent window (1 Mb), this strategy might ignore genes under selection but with strong LD to the top signal gene in the same region. For example, *TMEM247* and *ATP6V1E2* are located 42 kb and 104 kb downstream of *EPAS1*, respectively. Both genes harbor loci with strong selection signals (rs57720200, CMS = 14.29; rs4953388, CMS = 13.15). However, considering their strong LD between *EPAS1*, these two genes were excluded from our list (Additional file 2: Fig. S7A). Nevertheless, the 192 TSNGs identified in this study are a robust and high-confident list since only those genes with repeatable signals in multi-methods were included.

Among the list of 192 TSNGs, *EPAS1* and *EGLN1* remained in the top signals, consistent with previous genetic and functional studies [14, 62]. Importantly, among the top 10 TSNGs, we identified four novel genes, i.e., *TMEM132C*, *ATP13A3*, *SANBR*, and *KHDRBS2* (Fig. 3A). These four newly identified genes provide valuable clues to delineate the genetic basis of adaptation of cardio-pulmonary function in Tibetans. We speculate that *TMEM132C* and *ATP13A3* might be the candidate genes responsible for the better pulmonary function of Tibetans since they were previously shown associated with pulmonary pressure and ventilation [63–65]. *SANBR* is another gene associated with lung function based on previous reports [42]. We found a cluster of *SANBR* TSNSs were eQTLs in the lung (Additional file 2: Fig. S8), suggesting a functional potential for these TSNSs in Tibetans. *KHDRBS2* is an important member of PTK6 signaling and is associated with atrial septal defect [47, 48]. Abundant studies declared that *KHDRBS2* was a prognostic marker of lung cancer [66, 67], and large-scale population GWAS showed that *KHDRBS2* was associated with lung airflow (the FEV/FEC ratio) [47, 48].

As the vital organ for gas exchange of organisms, the lung plays an essential role in balancing the respiratory system and O_2_ uptake at high altitudes. Previous studies pointed that Tibetan indigenous have larger total lung capacity and larger vital capacity compared to the lowlanders [68, 69]; however, no relevant gene was reported. Here, we found 24 TSNGs involved in lung development and ventilation (Fig. 5), which serve as a candidate set for screening the causal variants responsible for adaptive lung traits in Tibetans.

Interestingly, we found that 26 TSNGs (13.5%) were associated with reproduction. It is known that hypoxia may cause preeclampsia and intrauterine growth restriction, which greatly increase the risk of stillbirth and infant mortality [70, 71]. Compared with lowlanders living at high altitudes, Tibetans show significantly lower neonatal mortality [57, 70]. Hence, the 26 identified reproduction-related TSNGs may contribute to the increased reproductive fitness of Tibetans.

Finally, the newly identified TESVs are potentially related to Tibetans’ adaptive traits. For example, the two TESVs in *PKHD1L1* and *ESRRB* are related to sleep quality and placental development, respectively [72–74]. Since structural variants involve relatively large fragments in the genome, they may play a role in the genetic adaptation of Tibetans, which call for further studies.

## Conclusions

In conclusion, we generated the large-scale WGS data of Tibetans and provided the population-specific reference panel for Tibetan populations. We identified a high-confident set of genes (192 TSNGs) with signals of positive selection. These genes likely function in multiple organs/systems in the body with polygenic and pleiotropic effects, and they may work together to shape the adaptive traits in Tibetans. These findings demonstrate the great value and potential of large-scale WGS data for human population studies.

## Methods

### Samples and sequencing

A total of 1064 subjects were recruited in a hospital at Lhasa in Tibetan Autonomous Region, China (elevation: 3650 m above sea level). These recruited Tibetans are unrelated females (from the Obstetrics and Gynecology Department of the hospital), and they were from 83 different geographic locations (altitude range: 2300–4900 m). Written informed consent was obtained from each subject. The protocol of this study was reviewed and approved by the Internal Review Board of Kunming Institute of Zoology, Chinese Academy of Sciences (Approval ID: SMKX-20160311–45). Blood samples were collected, genomic DNA were extracted by Prefilled Blood DNA Kit–DUO (KFRPD-801212), and WGS was performed on the Illumina Nova-seq platform with an average of 40 Gb (11.8 × depth) data per individual (Fig. 1B). Prior to sequencing and analysis, all samples were stripped of personal identifiers to protect privacy. All procedures were in accordance with the ethical standards of the Responsible Committee on Human Experimentation.

### Data processing

#### QC and alignment

The raw reads generated by sequencing were subjected to quality control (QC) using FastQC (https://www.bioinformatics.babraham.ac.uk/projects/fastqc/) and the reads with low quality were removed. The reads (pass QC) were mapped to the human reference genome GRCh37(hs37d5) using the BWA-MEM algorithm of the bwa software (bio-bwa.sourceforge.net/bwa). Optical and PCR duplicate reads were marked with Picard MarkDuplicates (https://broadinstitute.github.io/picard/) and reads were sorted by SAMtools (http://www.htslib.org/, v.0.1.19).

#### Variant calling

After multi-step QCs, the alignments in the combined BAM file were locally realigned and recalibrated, and variants were called for all 1064 samples together using the Genome Analysis Toolkit (GATK, https://gatk.broadinstitute.org, v3.6). The Best Practices Workflows of variant discovery by standard GATK was used. We called variants per sample using the HaplotypeCaller module of GATK, and then, we performed joint genotyping and applied variant quality score recalibration (VQSR) filtering to produce the final multi-sample callset with the desired balance of precision and sensitivity. Finally, we identified 34,699,240 variants for the 1064 Tibetan individuals.

#### Individual QC

The initial dataset consisted of 1064 individuals and was subjected to additional QCs: (1) 5 individuals were removed with elevated missing rates or outlying heterozygosity rate, and we excluded the individuals with a genotype failure rate ≥ 0.03 and heterozygosity rate ± 3 standard deviations from the mean; (2) 2 duplicates and 56 ancestry-mix samples were excluded. In the end, we retained 1001 individuals who passed the individual QC. EIGENSOFT (https://reich.hms.harvard.edu/software, v.7.2.1) was used to conduct the principal component analysis (PCA) analysis. In this step, we considered the influence of LD and used PLINK to prune the data (–indep-pairwise 50 5 0.2).

#### Variant QC

For the 1001 clean data after individual QC, VCFtools (http://vcftools.sourceforge.net/, v.0.1.16) was used to divide the variants into SNVs (29,878,184) and INDELs (4,821,056), and the following steps were employed: (1) exclude the singleton variants, (2) exclude the SNVs with missing genotype data (–geno) larger than 3%, and (3) exclude the SNVs with an extra deviation of Hardy–Weinberg test (–hwe) *p* < 1e − 10. Finally, 28,189,099 SNVs remained.

### Variant annotations

After the QCs, we calculated the allele frequency and only kept biallelic SNVs for downstream analyses. We identified 28.2 million biallelic SNVs, and we made functional annotations of these SNVs using ANNOVAR (https://annovar.openbioinformatics.org/), VEP (https://useast.ensembl.org/Tools/VEP), and SnpEff (http://pcingola.github.io/SnpEff/). The shared SNVs among the three different annotation tools were considered high-impact variants. Also, we annotated the SNVs by clinical importance using ClinVar Database (https://www.ncbi.nlm.nih.gov/clinvar/, v.20181028) and by GWAS hits using the GWAS catalog (https://www.ebi.ac.uk/gwas/, v.2019–10-14). Additionally, the conservation levels of variants were evaluated using GERP, phyloP, PhastCons, Eigen, and CADD scores (https://cadd.gs.washington.edu/download, v.2.1.1).

### DAF and F_ST_

The derived allele frequency (DAF) of the whole-genome SNVs was calculated by PLINK (https://www.cog-genomics.org/plink2,v.2) (–freq) after identifying the ancestral alleles. We used *F*_ST_ to estimate the pairwise genetic distance between the Tibetan population and other populations of 1KGP3. *F*_ST(Tibetan-Han)_ was calculated by PLINK (–fst) (Additional file 2: Fig. S1D).

### Hardy–Weinberg equilibrium (HWE) score

The HWE score was calculated by PLINK (–hardy). We screened the SNVs that significantly deviated from HWE (*p* < 1e − 10). The correlation between HWE and DAF of SNVs was calculated using R (v.4.1).

### LD decay

LD decay of Tibetan and other populations was evaluated by PopLDdecay software (https://github.com/BGI-shenzhen/PopLDdecay) with the default parameters.

### Construction of the 1KTGP reference panel

The 28.2 million SNVs were used in the construction of the 1KTGP reference panel. The Shapeit2 (https://mathgen.stats.ox.ac.uk/genetics_software/shapeit,v.217) and IMPUTE2 (v.2.3.1) (https://mathgen.stats.ox.ac.uk/impute/impute,v.2.3.1) were used for haplotype phasing, and we imputed genotypes for the published array data containing 3008 individuals by reference panel 1KGP3 and 1KTGP, respectively. The imputation was performed with the command *impute2 -use_prephased_g -Ne 20,000 -iter 30 -align_by_maf_g -os 0 1 2 3 -seed 1,000,000*.

### Detection of genomic signatures of positive selection

To detect genome-wide signatures of positive selection in Tibetans, the haplotype-based methods (XPEHH and iHS), the allele frequency-based methods (*F*_ST_, ΔDAF, Tajima’s *D* test, and Fay and Wu’s *H* test), and the likelihood method (XPCLR) were employed. The CMS score of each variant was estimated by combining four statistics (XPEHH, iHS, *F*_ST_, and ΔDAF) [28]. We keep the four world populations (414 individuals) as reference populations (Han Chinese, Japanese, Europeans, and Africans) from 1KGP3. After QCs, 13,124,482 known ancestral allele SNVs remained (ancestral alleles identified to use the database proved by ensemble, version = 100; url = https://e100.ensembl.org/homo_sapiens), finally merging with 1001 Tibetans clean SNVs, and 9,672,742 common SNVs remained to detected the positive selection using different methods.

#### XPEHH and iHS

Selscan [75] was used to calculate the XPEHH and iHS of the genome-wide variants. Whole-genome level normalization was conducted, and 74,929 SNVs were excluded due to EHH decayed below 0.05.

#### ΔDAF

By identifying the ancestral alleles, we used PLINK to calculate the derived allele frequency (DAF) of the genomic variants, and then the DAF difference between Tibetans and other populations was calculated as ΔDAF.

#### CMS score

The scores of the composite of multiple signals (CMS) of the genomic variants were calculated by combining *F*_ST_, **Δ**DAF(Tibetans-Han), XPEHH, and iHS. The CMS score for each variant was calculated using the formula from the previous study [15].

We calculated the CMS scores of 9,597,813 SNVs, and the top 1‰ SNVs (9598) with the highest CMS scores were considered the candidate SNVs. We further filtered the 9598 SNV set by only keeping the SNVs showing consistent Tibetan-specific enrichment when comparing their frequencies in Tibetans with those in four other reference populations (CHB, JPT, CEU, and YRI from 1KGP3). The remaining 4320 SNVs were defined as Tibetan selection-nominated SNVs (TSNSs). Next, we performed the LD-based clumping to identify independent signal regions (*r*^2^ ≦ 0.2 and clump window size of 500 kb). We identified 236 independent signal regions with significant positive selection in Tibetan populations, involving 379 genes. The nearest gene of the top TSNS in each independent region (1-Mb block) was designated as the Tibetan selection-nominated gene (TSNG), and a gene region is defined by the gene body plus the upstream and downstream flanking 5-kb segments. In total, 192 TSNGs were characterized.

### Functional annotation of the 192 TSNSs

The variant types were provided by VEP according to the sequence ontology. To explore the potential effects of TSNSs on gene expression, we downloaded and extracted all significant variant-gene pairs of 49 tissues (*p* < 0.05) from the Genotype-Tissue Expression (GTEx) database (v.8). GTEx Portal (https://www.gtexportal.org/home/) and FIVEx (https://fivex.sph.umich.edu/) were used for visualization [76, 77].

### Enrichment analysis

Functional enrichment, including pathway (KEGG), biological process (GO), and disease (disGeNET) annotations of TSNGs were analyzed by Metascape (https://metascape.org/) [78]. Besides, we applied a context-specific regulation for variants (SpecVar)-based method [79] to evaluate the enrichment of TSNSs in the underlying regulatory networks across diverse human cellular/tissue contexts. We overlapped our TSNSs to phenotypic traits data from the GWAS catalog (Additional file 1: Table S14). Fisher’s exact test was performed to test the enrichment significance of phenotypic traits, and the *p*-value was corrected by the Bonferroni method.

In order to link the TSNGs to the body parts they potentially affect, we utilized Gene ORGANizer [51] combining the GWAS catalog database and Mouse Genome Informatics (MGI) database to establish the connections between TSNGS and organ systems.

### Structural variant genotyping

Based on a high-confident breakpoint set of 17,900 SVs from our previous study [21], we conducted genotyping of these SVs in bam files of our 1001 Tibetan WGS data using the SVTyper [80] and Paragraph [81] tools. SVTyper filtered out the SVs with support reads < 5 and low variant quality (QUAL < 10) (Additional file 2: Fig. S2A). Paragraph filtered out the SVs based on the following indexes: (1) one or more breakpoints have abnormal depth (BP_DEPTH), (2) one genotype was missing (BP_NO_GT), (3) no valid genotypes from breakpoints (NO_VALID_GT), and (4) breakpoints gave different genotypes (CONFLICT) (Additional file 2: Fig. S2B). SVs passing both SVTyper and Paragraph were merged to generate the final SV set. After QC, we successfully genotyped 9490 SVs (Additional file 1: Table S16).

An SV with high-frequency divergences (> 20%) between Tibetans and other global populations was designated as a TESV. Similar to the gene assignment for TSNSs, the nearest gene of a TESV was taken as the assigned gene.

### Polygenic selection analysis

We employed the PolyGraph tool to identify signatures of polygenic selection in Tibetans [56]. We retrieved a set of SNVs (3643 SNVs) associated with blood hemoglobin level (HGB) using summary statistics from the large-scale GWAS meta-analysis of UK Biobank. To obtain a list of independent SNPs, we removed those SNVs in LD (*r*^2^ > 0.2 in 1KGP CEU). Finally, we obtained 2031 independent HGB-associated SNVs (including 7 SNVs located in the *EPAS1*gene region). At the same time, we generated a set of 799,055 independent SNVs of Tibetans as the control panel. These SNVs are unlinked SNVs, and they are not significantly associated with the HGB level and have lower LD (*r*^2^ < 0.2) in the 1001 Tibetan WGS data. We used the default input parameters, and Bonferroni correction was applied to the adjustment of multiple tests.

### Archaic introgression analysis

ArchaicSeeker2.0 [35] and SPrime [36] were used to search for the introgression regions of archaic hominins. In accordance with the protocol [35, 82], we downloaded the outgroup data (Chimpanzee, https://drive.google.com/drive/folders/115LSXmYDlitNKDO58SgxbEYlNd4EG1WK?usp=sharing) and archaic hominins data (Altai Denisovans, http://cdna.eva.mpg.de/neandertal/altai/Denisovan and Altai Neanderthal, http://cdna.eva.mpg.de/neandertal/altai/AltaiNeandertal. The SNV QC was performed according to the protocol. In the SPrime analysis, for identifying the introgression regions of Altai Neanderthals, the following steps were performed: (1) exclude the regions with matched site < 30 and (2) exclude the region with matching rate < 60% and matching rate of Altai Denisovans > 40%. For Altai Denisovans, similar steps were used, including (1) excluding the region with matched site < 30 and (2) excluding the region with a matching rate < 40% and a matching rate of Altai Neanderthal > 30%.

## Supplementary Information

**Additional file 1: Table S1.** The previous studies of Tibetans. **Table S2.** The reported genes under selection in Tibetans. **Table S3.** Sample information in this study. **Table S4.** Variant annotations. **Table S5.** Variant types. **Table S6.** Summary of TSNGs. **Table S7.** Summary of TSNSs. **Table S8.** Enrichment results using GeneORGANizer. **Table S9.** The enrichment results using *specvar*. **Table S10.** The enrichment result using *metascape*. **Table S11.** The enrichment result using DisGeNET. **Table S12.** The enrichment results based on GWAS catalog. **Table S13.** The enrichment results based on MGI database.** Table S14.** The Tibetan-enriched SVs. **Table S14.** The Tibetan-enriched SVs. **Table S15. **The introgression regions of archaic hominins in Tibetans. **Table S16. **The genotyping information of SVs.

**Additional file 2:** **Fig. S1.** Genetic architecture of Tibetans based on the 1,001 WGS data. **Fig. S2. **Tibetan SV analysis based on the 1,001 WGS data.** Fig. S3. **Evaluation of HWE and imputation efficiency using 1KTGP. **Fig. S4. **Genomic signatures of positive selection in Tibetans. **Fig. S5.** Distributions of frequency difference of the 9,508 SVs between Tibetans and other populations (lowlanders). **Fig. S6. **The results of archaic introgression in Tibetans. **Fig. S7.** Regional plots of the CMS scores and recombination rates of six TSNGs in the top 10 list with previously reported selective signals. **Fig. S8. **The eQTL map of the top TSNS rs1627608 in *SANBR* based on the GTEx database. **Fig S9.** The PloyGraph diagrams of the trait-associated variants that show patterns of polygenic selection on the HGB level in Tibetans. **Fig. S10. **Natural selection test of *MTHFR* (A) and *PPARA* (B). No significant positive selection signals in these two genomic regions are detected. **Fig. S11. **Natural selection test of the two previously reported TSNGs: *VDR* (A) and *DNMBP* (B). The significant thresholds for all statistics are marked by the dashed lines in red.

**Additional file 3.** Review history.

## Data Availability

The whole-genome sequencing data generated in this study have been deposited to the Genome Sequence Archive under the accession number (PRJCA007843) (https://bigd.big.ac.cn/gsa-human/browse/HRA001809) [83].
