# Supplementary material for: Large-scale genome sequencing redefines the genetic footprints of high-altitude adaptation in Tibetans
Source: Genome Biol. 2023 Apr 13;24:73. doi: 10.1186/s13059-023-02912-1 (PMC10099689; doi:10.1186/s13059-023-02912-1)
Supplement: Supplementary file 2 — Additional file 2: Fig. S1. Genetic architecture of Tibetans based on the 1,001 WGS data. Fig. S2. Tibetan SV analysis based on the 1,001 WGS data. Fig. S3. Evaluation of HWE and imputation efficiency using 1KTGP. Fig. S4. Genomic signatures of positive selection in Tibetans. Fig. S5. Distributions of frequency difference of the 9,508 SVs between Tibetans and other populations (lowlanders). Fig. S6. The results of archaic introgression in Tibetans. Fig. S7. Regional plots of the CMS scores and recombination rates of six TSNGs in the top 10 list with previously reported selective signals. Fig. S8. The eQTL map of the top TSNS rs1627608 in SANBR based on the GTEx database. Fig S9. The PloyGraph diagrams of the trait-associated variants that show patterns of polygenic selection on the HGB level in Tibetans. Fig. S10. Natural selection test of MTHFR (A) and PPARA (B). No significant positive selection signals in these two genomic regions are detected. Fig. S11. Natural selection test of the two previously reported TSNGs: VDR (A) and DNMBP (B). The significant thresholds for all statistics are marked by the dashed lines in red. [file 13059_2023_2912_MOESM2_ESM.pdf]

Large-scale genome sequencing relocates the genetic footprints of high-altitude adaptation in Tibetans

Wangshan Zheng[1,5]*, Yaoxi He[1]*, Yongbo Guo[1,5]*, Tian Yue[1,5]*, Hui Zhang[1,5]*, Jun Li[2]*, Jingxin Cao[2], Caijuan Bai[6], Bin Wang[2], Bin Zhou[1,5], Xuerui Zeng[1,5], Liya Li[1], Li Chen[2], Chunxia Li[2], Hongyan Li[2], Baimakangzhuo[3], Chaoying Cui[3], Xuebin Qi[1#], Ouzhuluobu[2,3#], Bing Su[1,4#]

[1]State Key Laboratory of Genetic Resources and Evolution, Kunming Institute of Zoology, Chinese Academy of Sciences, Kunming 650223, China;

[2]Fukang Obstetrics, Gynecology and Children Branch Hospital, Tibetan Fukang Hospital, Lhasa 850000, China;

[3]High Altitude Medical Research Center, School of Medicine, Tibetan University, Lhasa 850000, China;

[4]Center for Excellence in Animal Evolution and Genetics, Chinese Academy of Sciences, Kunming 650223, China;

[5]Kunming College of Life Science, University of Chinese Academy of Sciences, Beijing 100101, China;

[6]People's Hospital of Gansu Province, Lanzhou 730000, China

*These authors contributed equally to this work.

#Corresponding authors: Bing Su, Ouzhuluobu or Xuebin Qi

Email: sub@mail.kiz.ac.cn, tbciyang@163.com, qixuebin1973@163.com.

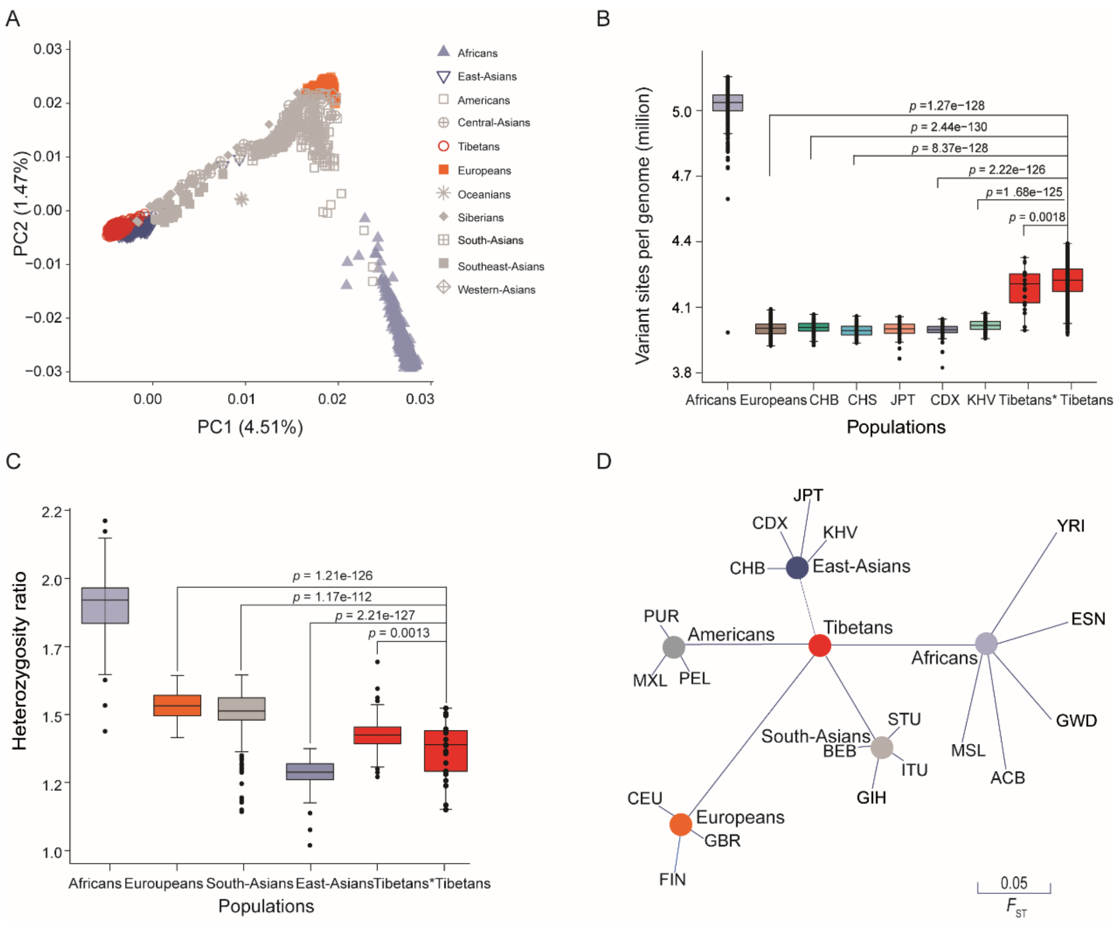

Fig. S1 Genetic architecture of Tibetans based on the 1,001 WGS data. A. Genome-wide PCA plot of the 1,001 Tibetans and global populations. **B.** Estimation of variant sites versus reference genome in Tibetans and representative global populations. **C.** Comparison of heterozygosity ratios among Tibetans and other populations. **D.** Genetic divergence (estimated by pairwise F_{ST}) between the Tibetan populations and world-wide populations. Tibetan: the 1,001 Tibetans in this study; Tibetan*: the 33 Tibetans from the previous study (Deng et al. 2019).

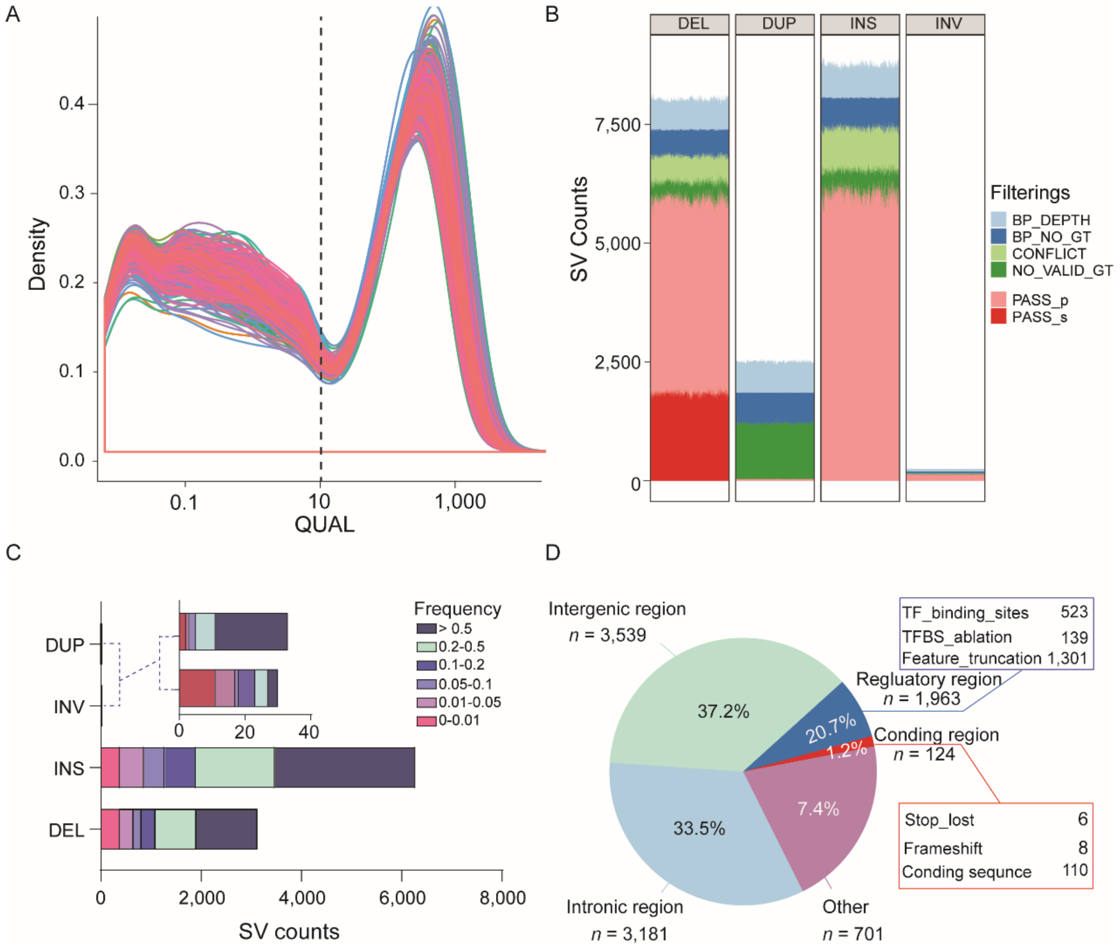

Fig. S2 Tibetan SV analysis based on the 1,001 WGS data. A. Distribution of SV quality (QUAL) by SVTyper. QC of SVTyper was conducted by QUAL > 10 (dashed line). **B.** SV counts from various filters by Paragraph and SVTyper. The SV numbers passing QC by Paragraph and SVType are denoted as PASS_p and PASS_s, respectively. **C.** The SV frequency spectrum of all identified SVs. **D.** Functional annotations of the 9,508 SVs.

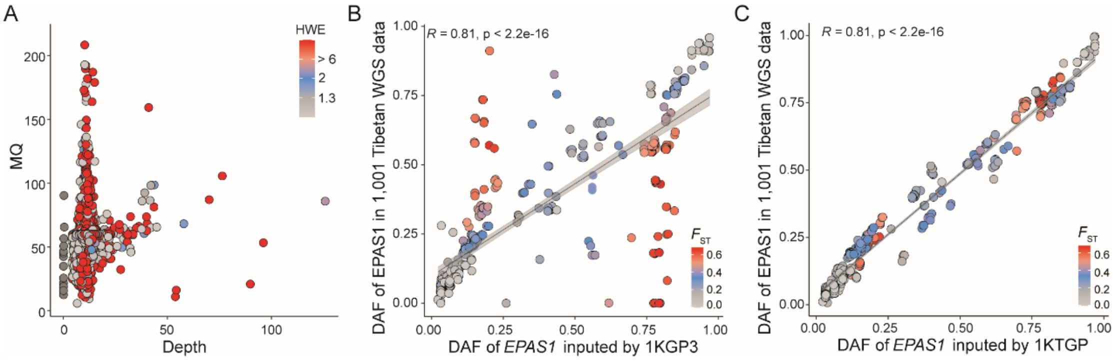

Fig. S3 Evaluation of HWE and imputation efficiency using 1KTGP. A. Correlation between depth and MQ (mapping quality) of the whole-genome SNVs underlying different HWE deviations in the 1,001 Tibetans. The pattern reveals that those SNVs with extra-deviation of HWE have good quality with high MQ and depth. **B.** The correlation of DAF of the *EPAS1* SNVs from the 1,001 Tibetan WGS data and from the 3,008 Tibetan array data imputed by 1KGP3. **C.** The correlation of DAF of the *EPAS1* SNVs from the 1,001 Tibetan WGS data and from the 3,008 Tibetan array data imputed by IKTGP.

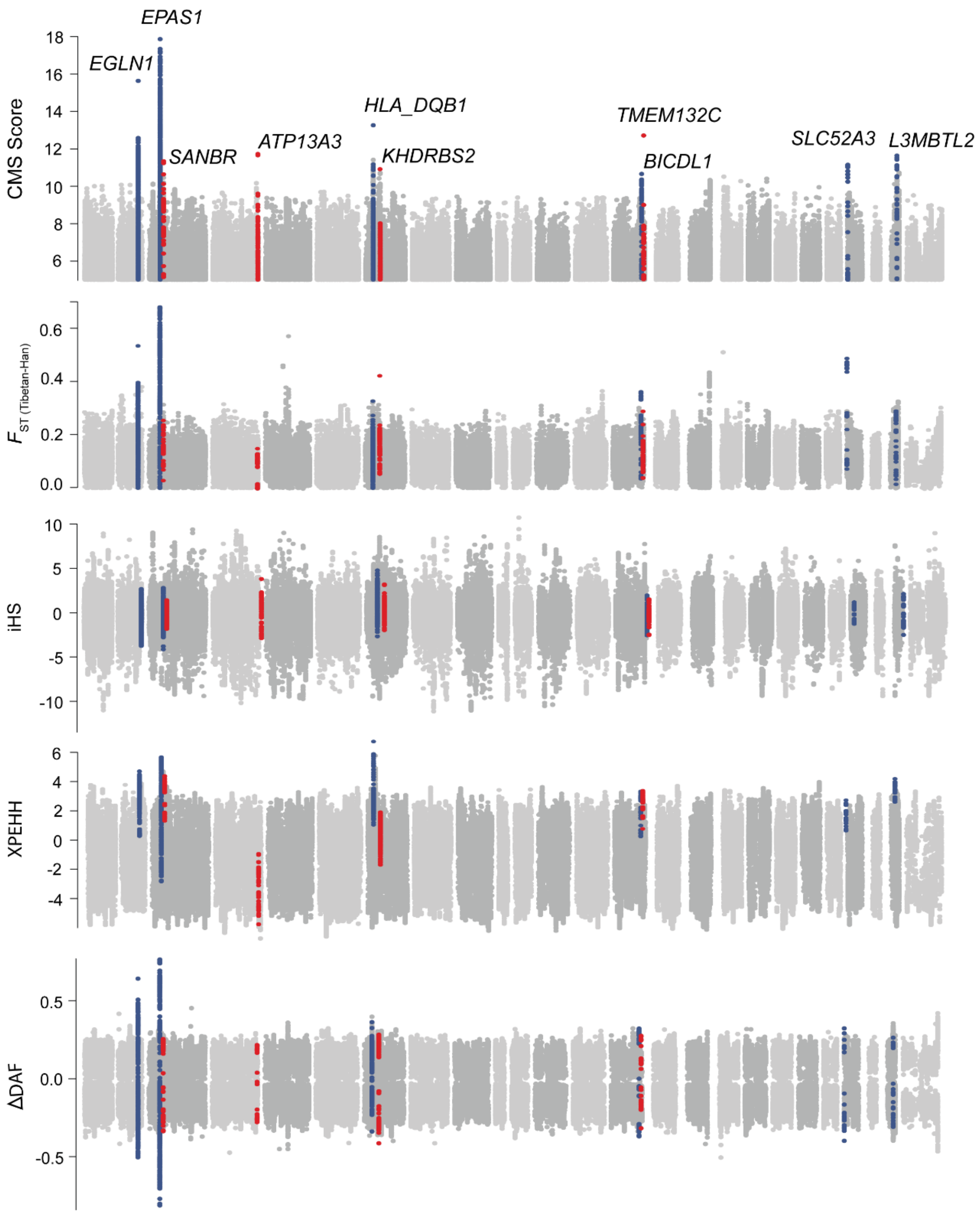

Fig. S4 Genomic signatures of positive selection in Tibetans. The distribution of the top 10 TSNGs in Tibetans were marked in red (newly-identified genes) and blue (reported genes) dots.

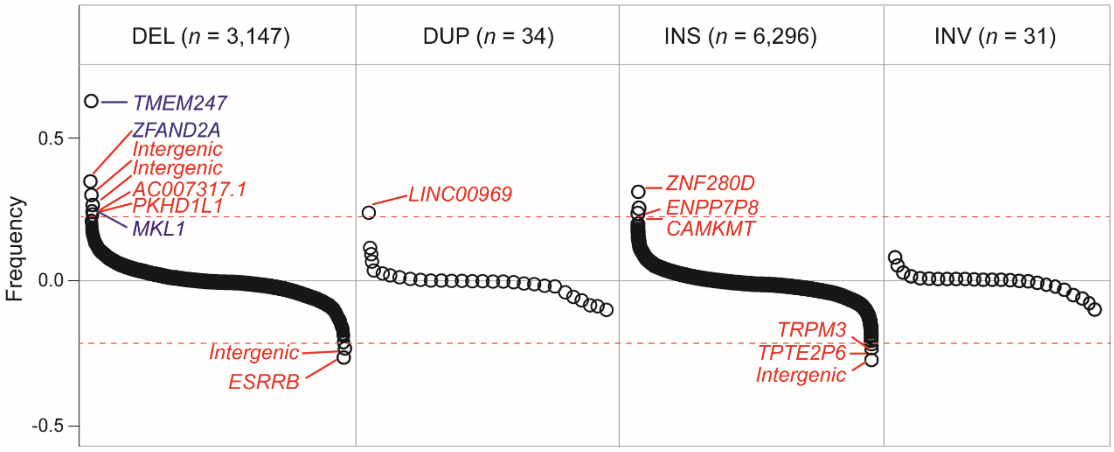

Fig. S5 Distributions of frequency difference of the 9,508 SVs between Tibetans and other populations (lowlanders). The newly identified Tibetans-enriched SVs (TESVs) in this study and the previously reported TESVs were marked in red and blue, respectively.

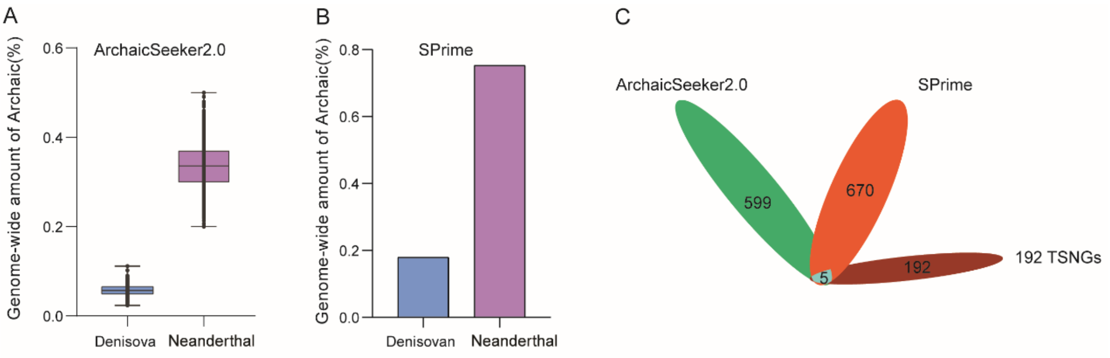

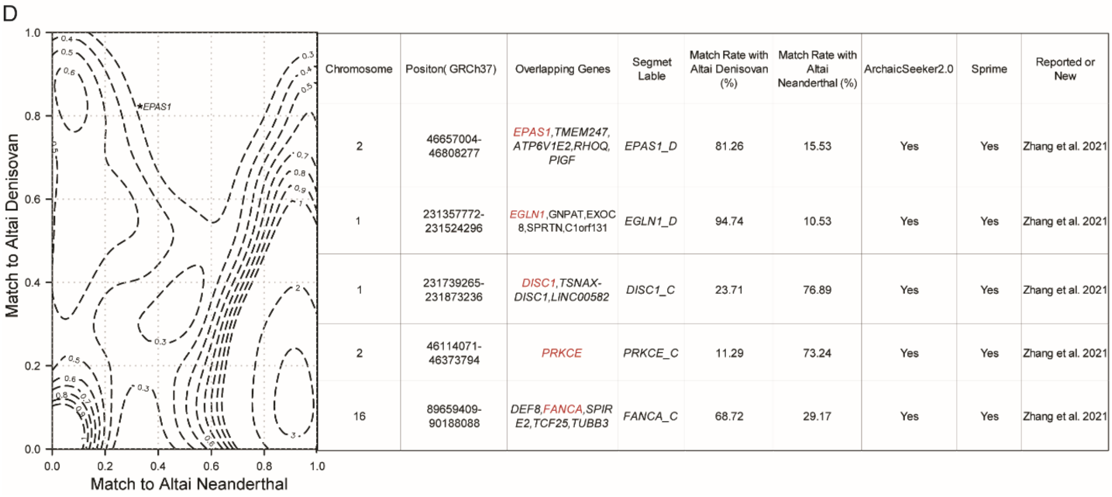

Chromosome	Positon(GRCh37)	Overlapping Genes	Segmet Lable	Match Rate with Altai Denisovan (%)	Match Rate with Altai Neanderthal (%)	ArchaicSeeker2.0	Sprime	Reported or New
2	46657004-46808277	*EPAS1,TMEM247, ATP6V1E2,RHOQ, PIGF*	*EPAS1_D*	81.26	15.53	Yes	Yes	Zhang et al. 2021
1	231357772-231524296	*EGLN1*,GNPAT,EXOC 8,SPRTN,C1orf131	*EGLN1_D*	94.74	10.53	Yes	Yes	Zhang et al. 2021
1	231739265-231873236	*DISC1,TSNAX-DISC1,LINC00582*	*DISC1_C*	23.71	76.89	Yes	Yes	Zhang et al. 2021
2	46114071-46373794	*PRKCE*	*PRKCE_C*	11.29	73.24	Yes	Yes	Zhang et al. 2021
16	89659409-90188088	*DEF8,FANCA,SPIR E2,TCF25,TUBB3*	*FANCA_C*	68.72	29.17	Yes	Yes	Zhang et al. 2021

Fig. S6 The results of archaic introgression in Tibetans. **A.** The ratios of Denisovan and Neanderthal introgression ratios in Tibetans by ArchaicSeeker 2.0; **B.** The ratios of Denisovan and Neanderthal introgression ratios in Tibetans by SPrime. **C.** The Venn diagram showing the identified introgression regions in the Tibetan genomes, detected by two commonly use tools, and their overlaps with the192 TSNGs. Only 5 positively selected genes show archaic introgression; **D.** The genome-wide match rates with the archaic hominins. The plot shows the putatively archaic introgression in *EPAS1* and the density distribution of the match rate to Denisovans or Neanderthals, inferred by the SPrime program using Africans (YRI) as the outgroup (left panel). The 5 introgression regions overlapped with the positively selected genes (right panel), and the labels of the segments (with "-U/C/D") correspond to upstream, core and downstream of the gene regions.

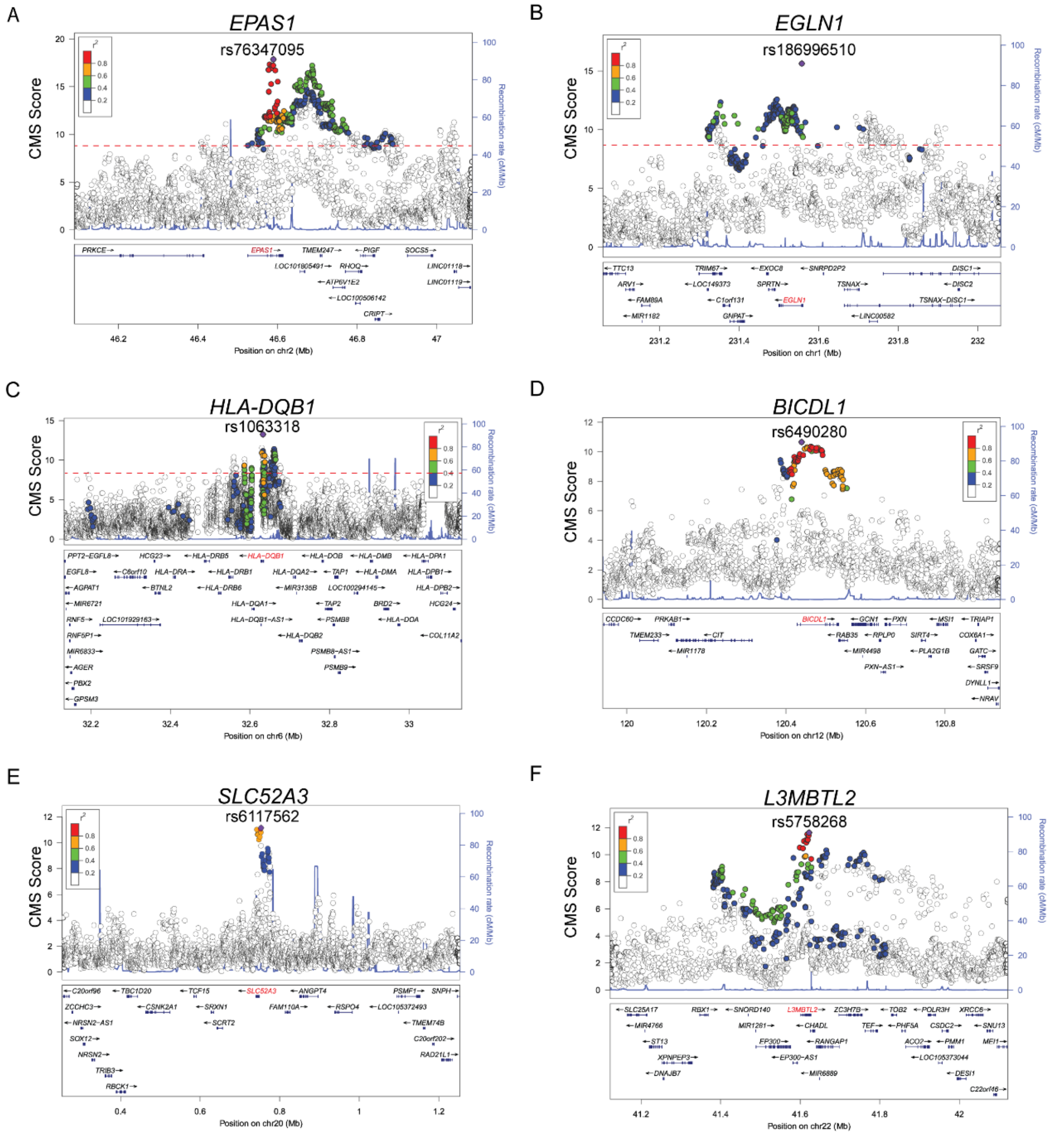

Fig. S7 Regional plots of the CMS scores and recombination rates of six TSNGs in the top 10 list with previously reported selective signals. (A-F): *EPAS1*, *EGLN1*, *HLA-DQB1*, *BICDL1*, *SLC52A3* and *L3MBTL2*. The SNV ID and the *p* values of the most significant peak SNVs are labeled. The LD (measured by r^2) between the peak SNV and the other SNVs were estimated using the 1KGTP haplotypes, and coded in colors.

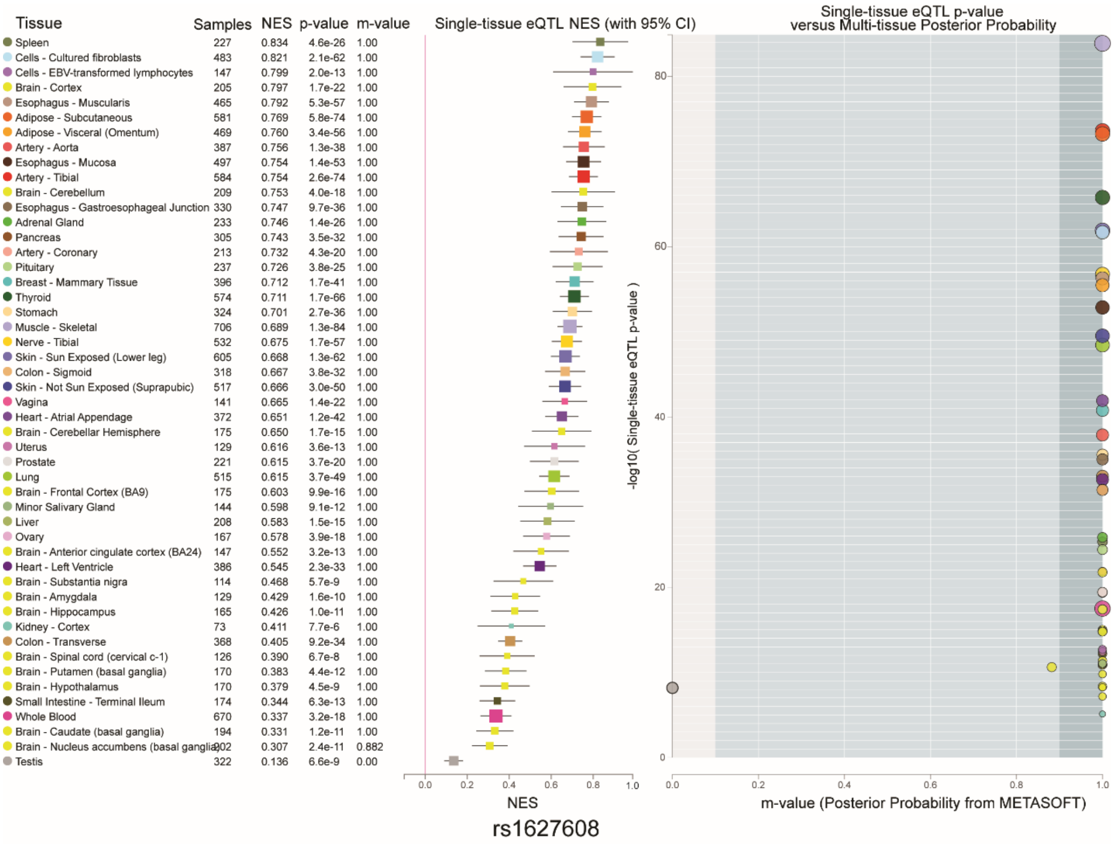

Tissue	Samples	NES	p-value	m-value
Spleen	227	0.834	4.6e-26	1.00
Cells - Cultured fibroblasts	483	0.821	2.1e-62	1.00
Cells - EBV-transformed lymphocytes	147	0.799	2.0e-13	1.00
Brain - Cortex	205	0.797	1.7e-22	1.00
Esophagus - Muscularis	465	0.792	5.3e-57	1.00
Adipose - Subcutaneous	581	0.769	5.8e-74	1.00
Adipose - Visceral (Omentum)	469	0.760	3.4e-56	1.00
Artery - Aorta	387	0.756	1.3e-38	1.00
Esophagus - Mucosa	497	0.754	1.4e-53	1.00
Artery - Tibial	584	0.754	2.6e-74	1.00
Brain - Cerebellum	209	0.753	4.0e-18	1.00
Esophagus - Gastroesophageal Junction	330	0.747	9.7e-36	1.00
Adrenal Gland	233	0.746	1.4e-26	1.00
Pancreas	305	0.743	3.5e-32	1.00
Artery - Coronary	213	0.732	4.3e-20	1.00
Pituitary	237	0.726	3.8e-25	1.00
Breast - Mammary Tissue	396	0.712	1.7e-41	1.00
Thyroid	574	0.711	1.7e-66	1.00
Stomach	324	0.701	2.7e-36	1.00
Muscle - Skeletal	706	0.689	1.3e-84	1.00
Nerve - Tibial	532	0.675	1.7e-57	1.00
Skin - Sun Exposed (Lower leg)	605	0.668	1.3e-62	1.00
Colon - Sigmoid	318	0.667	3.8e-32	1.00
Skin - Not Sun Exposed (Suprapubic)	517	0.666	3.0e-50	1.00
Vagina	141	0.665	1.4e-22	1.00
Heart - Atrial Appendage	372	0.651	1.2e-42	1.00
Brain - Cerebellar Hemisphere	175	0.650	1.7e-15	1.00
Uterus	129	0.616	3.6e-13	1.00
Prostate	221	0.615	3.7e-20	1.00
Lung	515	0.615	3.7e-49	1.00
Brain - Frontal Cortex (BA9)	175	0.603	9.9e-16	1.00
Minor Salivary Gland	144	0.598	9.1e-12	1.00
Liver	208	0.583	1.5e-15	1.00
Ovary	167	0.578	3.9e-18	1.00
Brain - Anterior cingulate cortex (BA24)	147	0.552	3.2e-13	1.00
Heart - Left Ventricle	386	0.545	2.3e-33	1.00
Brain - Substantia nigra	114	0.468	5.7e-9	1.00
Brain - Amygdala	129	0.429	1.6e-10	1.00
Brain - Hippocampus	165	0.426	1.0e-11	1.00
Kidney - Cortex	73	0.411	7.7e-6	1.00
Colon - Transverse	368	0.405	9.2e-34	1.00
Brain - Spinal cord (cervical c-1)	126	0.390	6.7e-8	1.00
Brain - Putamen (basal ganglia)	170	0.383	4.4e-12	1.00
Brain - Hypothalamus	170	0.379	4.5e-9	1.00
Small Intestine - Terminal Ileum	174	0.344	6.3e-13	1.00
Whole Blood	670	0.337	3.2e-18	1.00
Brain - Caudate (basal ganglia)	194	0.331	1.2e-11	1.00
Brain - Nucleus accumbens (basal ganglia)	202	0.307	2.4e-11	0.882
Testis	322	0.136	6.6e-9	0.00

Fig. S8 The eQTL map of the top TSNSs rs1627608 in *SANBR* based on the GTEx database.

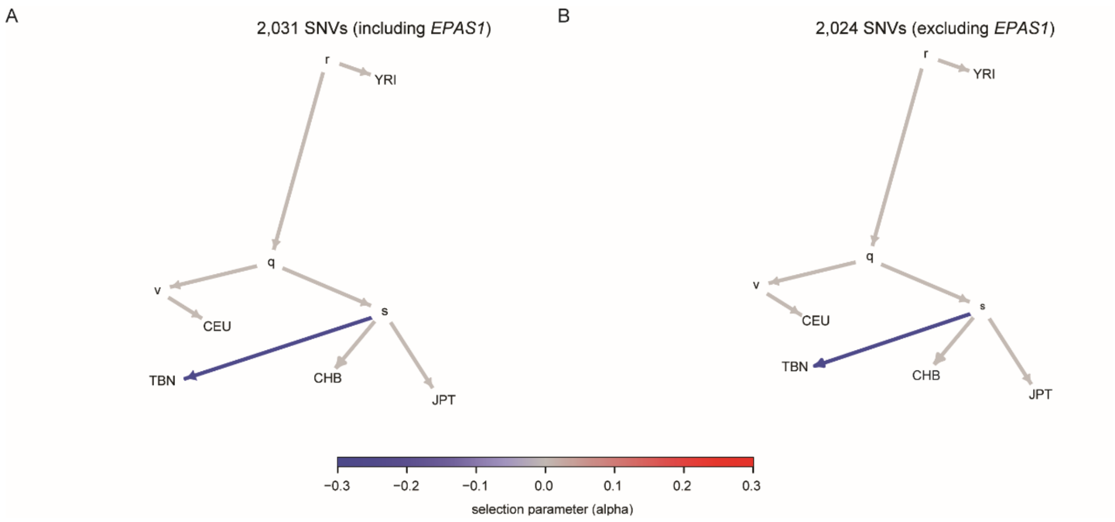

Fig S9. The PloyGraph diagrams of the trait-associated variants that show patterns of polygenic selection on the HGB level in Tibetans. **A.** The diagram for all the trait-associated variants (2,031 SNVs, including *EPAS1*); **B.** The diagram of the trait-associated variants by excluding the *EPAS1* variants (2,024 SNVs).

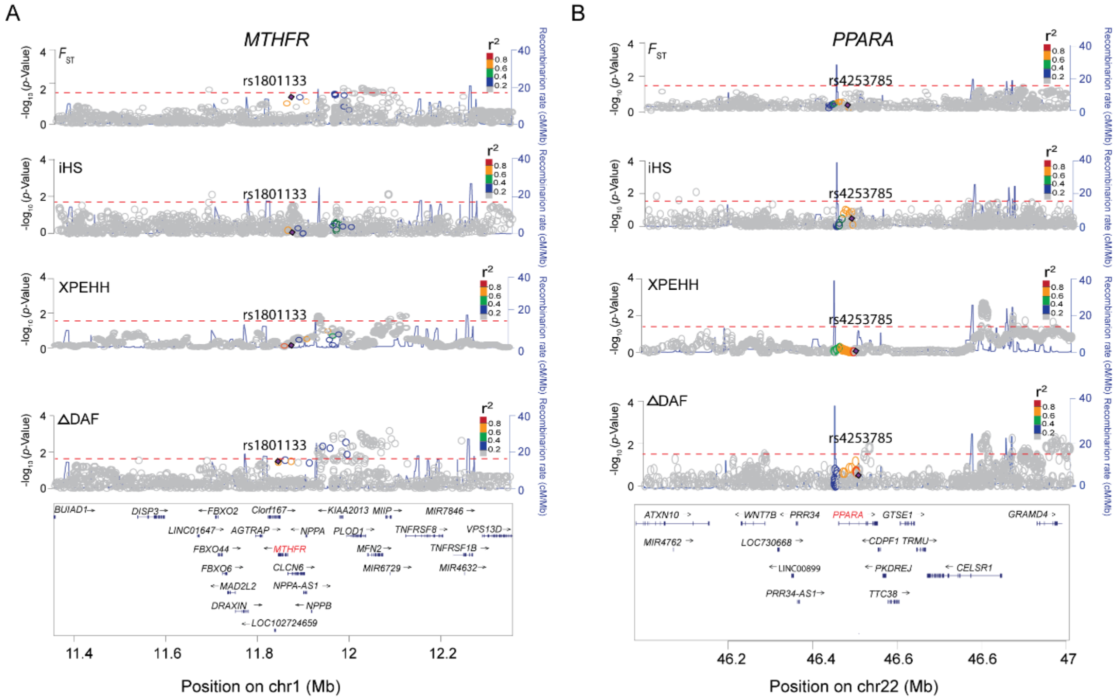

Fig. S10 Natural selection test of *MTHFR* (A) and *PPARA* (B). No significant positive selection signals in these two genomic regions are detected.

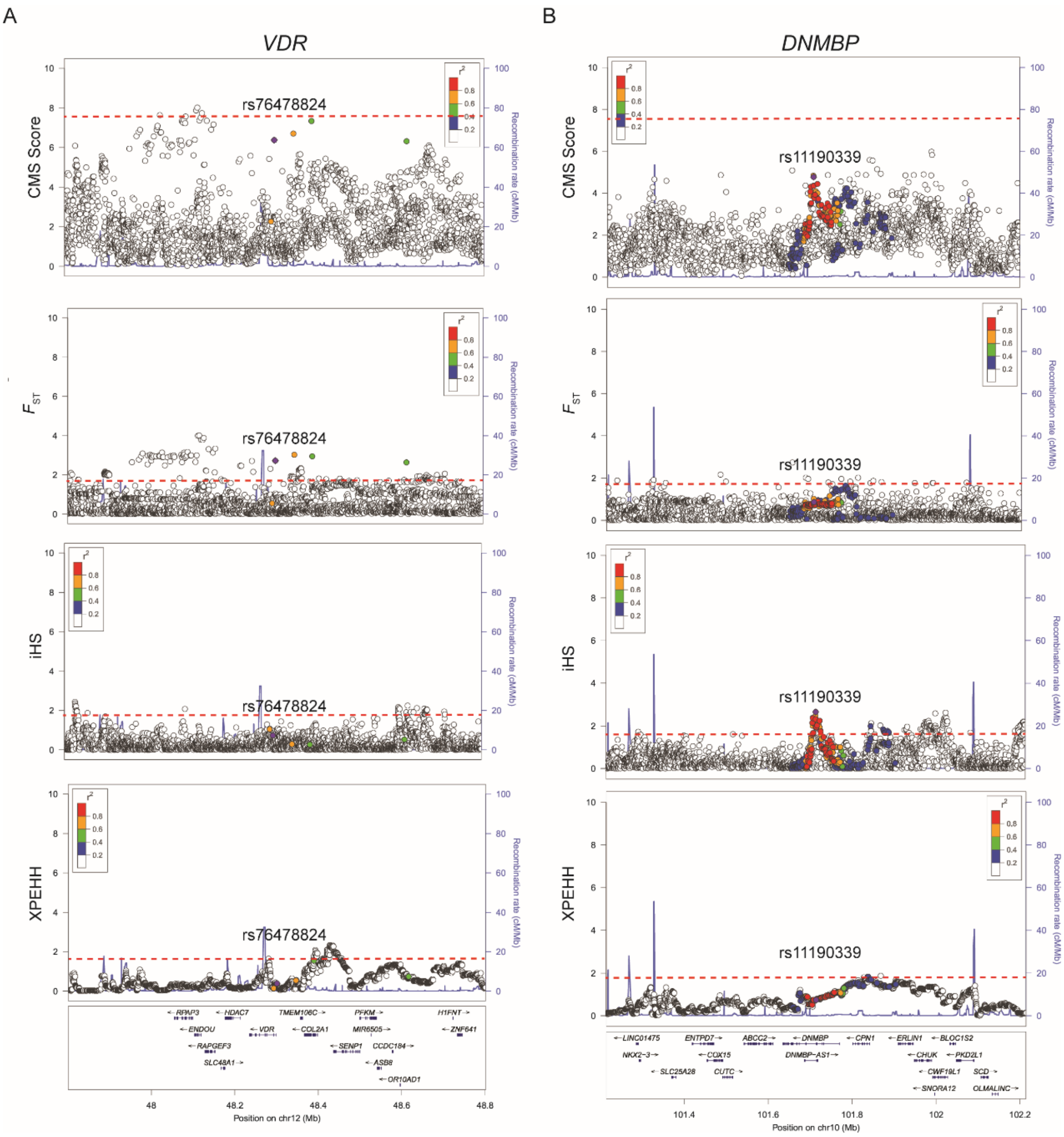

Fig. S11 Natural selection test of the two previously reported TSNGs: *VDR* (A) and *DNMBP* (B). The significant thresholds for each statistic are marked in red dashed lines.
