# Supplementary material for: Large-scale genome sequencing redefines the genetic footprints of high-altitude adaptation in Tibetans
Source: Genome Biol. 2023 Apr 13;24:73. doi: 10.1186/s13059-023-02912-1 (PMC10099689; doi:10.1186/s13059-023-02912-1)
Supplement: Supplementary file 3 — Additional file 3. Review history. [file 13059_2023_2912_MOESM3_ESM.docx]

**1^st^ round**

**Reviewer 1**

This is an exciting study that generates high-quality whole-genome sequencing (WGS) data for a large sample (N = 1001) from a unique population, the Tibetans. It generates a comprehensive set of genetic variants, including SNVs, INDELs, and SV. Over 30% of SNVs have not been previously reported in dbSNP. This high-quality dataset improves genetic imputation for Tibetan samples. The study also performed a series of tests for positive selection and summarized the signals with CMS approach. The newly identified genes under positive selection and their enriched pathways significantly advance our understanding of the genetic basis of adaptation to high altitude. This data is novel and opens up many research opportunities in the future. The data analysis approaches are fairly comprehensive and standard. This study replicated some previously well-known results, such as the top selection signals and the population relationships. Moreover, it included previous WGS Tibetan samples (N = 33) in some analyses (e.g., PCA, variant counts), and the results are consistent with those based on the new WGS samples (N = 1001). All these standard approaches and consistent observations brought confidence in the many novel results based on the new sequencing dataset. I support the publication of this study, but there are multiple places that need clarifications.

* Fig 2C is lacking explanations. It is unclear what the chromatograms are showing and what the p values are.
* In Fig 2D, it is unclear what the EPAS1 region r^2 refers to or how it was calculated. Usually, in regions under positive selection, there are extended haplotype homozygosity and thus slower LD decay. Why does the Tibeta_EPAS1 have a lower r^2 in comparison to other populations?
* It is unclear how were genes assigned to each independent genomic region with selection signals. Similarly, how were "lead genes" assigned? Also, how are genes assigned to the TESV? Are they the nearest genes to the SNV or SV?
* Page 12, line 12: "For example, the newly identified 322-bp intronic deletion in ENPP7P8 (encoding an ectonucleotide pyrophosphatase) is enriched (50%) in Tibetan populations, but relatively rare (<23%) in non-Tibetan populations. This gene is reportedly associated with sleep quality, a known physiological response at high altitude". However, based on Table S14, the only SV related to ENPP7P8 is an 82-bp insertion, with 58.2% frequency in Tibetan samples. On the other hand, a 322-bp deletion with 49.5% frequency in Tibetans is related to PKHD1L1. Please double check.
* Availability of data and materials. I could not find the associated data with the provided accession number "PRJCA007843". Please make sure the data is released before the formal publication.

Minor issues:

Page 14, line 4: "as the most oxygen-consuming orang". "orang" should be "organ".
Page 24, line 22: "IMPUT2" should be "IMPUTE2".
Page 26, line 17: "Feng et al. 2021, Paper in submission". If possible, please update the reference. Even a preprint is helpful.

**Reviewer 2**

Zheng et al. generated whole-genome sequence data from 1,064 individuals of indigenous Tibetans. After applying strict quality filters to the data, 1,001 genomes were subsequently used for scanning selection signals. More than one third of 35 million variants identified from this data set (30 million SNVs and 4.8 million INDELS) have not been previously annotated in any other human populations. Thus, this dataset provides an unprecedented opportunity to uncover novel genes associated with high-altitude adaptation. With the rigorous tests of selection, this study was able to provide a high-confident list of 4,320 variants across 192 genes (including 158 genes newly identified), which are enriched with the functions related to cardiopulmonary system, oxygen sensing, development, and reproductive viability. This is the largest whole-genome sequence data from Tibetans, as far as I am aware of, and the discoveries made in this study, in addition to the data produced, will be critical to improve our understanding on the long-standing mystery on high-altitude adaptation. Overall, I am convinced with the results presented in this manuscript and only have two major comments below.
1. The mean depth reported in this study is 11.8x, which may not be sufficient to confidently call SNVs with rare alleles. Particularly, if those alleles are uniquely present in the Tibetans, it can be very challenging to prove that they are not false positives. This could be less problem if all Tibetan individuals are pooled together for genotype calling. However, this does not seem to be the case in this study as the calling was done for each individual separately (see Page 22, Lines 25-26). How would the authors be confident with rare variant calling given that the majority of newly identified alleles are rare in the population (Fig. 1c)?
2. Given that the authors show the polygenic nature of high-altitude adaptation, it would be very interesting to see if there are any traits (e.g. blood hemoglobin level), based on UK BioBank or BioBank Japan, that have signatures of polygenic selection in this population. There are useful methods available for this analysis (https://github.com/FerRacimo/PolyGraph; Berg and Coop 2014 PLoS Genet).

Minor comments:
1. I am not sure if "relocate" is a right word to represent what was done in this study as my understanding is that this study is refining the selection signatures in Tibetans with the largest set of whole-genome sequence data.
2. Repeatability -> reproducibility?
3. Page 19, Lines 5-8: I don't think 1KGP3 data are biased towards European ancestry given that these are sequence data from global populations including African, Asian, and American ancestry as well. Any bias detected in this study is due to a lack of Tibetan ancestry in the panel for imputation.
4. Page 24, Line 22: IMPUT2 -> IMPUTE2
5. In Fig. S4, where can the results on SVs be found?

**Reviewer 3**

Zheng et al. whole genome sequenced 1001 Tibetan individuals. They detect selection using the CMS score, and do enrichment analysis to gain insight into what pathways are involved in this adaptation. I was excited to read this manuscript, since this is the largest set of whole genomes from a Tibetan sample, and Tibetans are an interesting population to study natural selection and demographic history. The reason is that Tibetans are adapted to high altitude, and several studies have suggested that these are genetic adaptations (not acclimatization), and studies have identified some of the loci underlying this adaptation. In addition, archaic introgression from a Denisovan-like population likely introduced (via introgression) the beneficial variation (at the EPAS1 gene) which is the strongest signal of selection in Tibetans.

This paper was easy to read, but the analyses and findings presented are underwhelming. The authors have access to the largest sample of Tibetans and focused their study on summarizing and cataloging variation, and it reads like a genome paper. The current findings presented do not provide a significant advance over previously published studies. In my opinion, the authors could have done more to make their study more interesting by studying the landscape of archaic introgression in this sample, and whether archaic variants influenced other genes involved in their high-altitude adaptation (in addition to EPAS1).

Some minor points:

Page 5, Line 8: "The genome-wide heterozygosity of Tibetans is 1.41" What are the units of 1.41?

Page 6, Line 3: "The known and novel variants are shown in red and blue respectively." The legend in the figure says the red are novel and blue are known…. Which does not make sense since we expect the novel ones to be low frequency.

Page 8, Lines 11-16: Why focus on these three SNPs? I assume that if FST is high, these SNPs have been identified in previous studies? Are these SNPs in any interesting genes that haven't previously been identified?

**Authors’ response**

This is an exciting study that generates high-quality whole-genome sequencing (WGS) data for a large sample (N = 1001) from a unique population, the Tibetans. It generates a comprehensive set of genetic variants, including SNVs, INDELs, and SV. Over 30% of SNVs have not been previously reported in dbSNP. This high-quality dataset improves genetic imputation for Tibetan samples. The study also performed a series of tests for positive selection and summarized the signals with CMS approach. The newly identified genes under positive selection and their enriched pathways significantly advance our understanding of the genetic basis of adaptation to high altitude. This data is novel and opens up many research opportunities in the future. The data analysis approaches are fairly comprehensive and standard. This study replicated some previously well-known results, such as the top selection signals and the population relationships. Moreover, it included previous WGS Tibetan samples (N = 33) in some analyses (e.g., PCA, variant counts), and the results are consistent with those based on the new WGS samples (N = 1001). All these standard approaches and consistent observations brought confidence in the many novel results based on the new sequencing dataset. I support the publication of this study, but there are multiple places that need clarifications.

Response: We appreciate the reviewer’s approval of publishing our study.

Fig 2C is lacking explanations. It is unclear what the chromatograms are showing and what the p values are?

Response: Following the reviewer’s suggestion, we added the explanations to Figure 2C in the legend.

In Fig 2D, it is unclear what the EPAS1 region r^2 refers to or how it was calculated.

Usually, in regions under positive selection, there are extended haplotype homozygosity and thus slower LD decay. Why does the Tibeta_EPAS1 have a lower r^2 in comparison to other populations?

Response: We realize that it is in fact impractical to label one specific gene (i.e. EPAS1) in this plot. Therefore, in the revised version, we removed the EPAS1 labels from the curves. Considering that this plot is intended to show a distinctive LD-decay pattern (calculated using genome-wide SNVs) of Tibetans compared to other world populations, in the revised version, we highlighted the regions with long LD decay (>100kb), in which Tibetans show a slower decay (reflected by the higher genome- wide r2 values) compared to other populations, an indication of extended haplotype homozygosity. Indeed, as pointed out by the reviewer, the EPAS1 region harbors higher LD and longer haplotype blocks due to positive selection, which was already reported in our previous study [R2]. We updated Figure 2D and its legend in the revised manuscript, and we added a brief description in the main text (Page 7, Line 11- 13).

It is unclear how were genes assigned to each independent genomic region with selection signals. Similarly, how were "lead genes" assigned? Also, how are genes assigned to the TESV? Are they the nearest genes to the SNV or SV?

Response: For the assignment of “lead genes”, as described in the method section, we firstly conducted LD-based clumping for the CMS scores of all the genome-wide SNVs and identified independent signal regions (r2 < 0.2 and a clump window size of 500 kb). Then we filtered the SNV set by only keeping the SNVs showing consistent Tibetan-specific enrichment when comparing their frequencies in Tibetans with those in four other reference populations (CHB, JPT, CEU and YRI from 1KGP3), and the filtered SNV set was considered as the Tibetan selection-nominated SNVs (TSNSs).

The nearest gene of the top TSNS in each independent region (1Mb block) was designated as the “lead gene” (i.e. the Tibetan selection-nominated gene, TSNG), and a gene region is defined by the gene body plus the upstream and downstream flanking 5 Kb segments (refer to the Methods section under the subtitle of “Detection of genomic signatures of positive selection” (Page 23, Line 11-14).

For the assignment of TESV, an SV with high frequency divergences (>20%) between Tibetans and other global populations was designated as the Tibetan-enriched SV (TESV). Similar to the gene assignment by SNVs, the nearest gene of a TESV was taken as the assigned gene (Page 24, Line 22-24).

Page 12, line 12: "For example, the newly identified 322-bp intronic deletion in ENPP7P8 (encoding an ectonucleotide pyrophosphatase) is enriched (50%) in Tibetan populations, but relatively rare (<23%) in non-Tibetan populations. This gene is reportedly associated with sleep quality, a known physiological response at high altitude". However, based on Table S14, the only SV related to ENPP7P8 is an 82-bp insertion, with 58.2% frequency in Tibetan samples. On the other hand, a 322-bp deletion with 49.5% frequency in Tibetans is related to PKHD1L1. Please double check.

Response: We thank the reviewer for pointing out this error. This was a mis- assignment of gene name. The correct gene should be “PKHD1L1”, and the other information is correct. The error is corrected in the revised manuscript (Page 10, Line 11-12).

Availability of data and materials. I could not find the associated data with the provided accession number "PRJCA007843". Please make sure the data is released before the formal publication.

Response: We understand the reviewer’s point. The data and materials will be released when our manuscript is officially published.

Minor issues:

Page 14, line 4: "as the most oxygen-consuming orang". "orang" should be "organ".

Response: This has been corrected accordingly (Page 14, Line 5).

Page 24, line 22: "IMPUT2" should be "IMPUTE2".

Response: This has been corrected accordingly (Page 22, Line 4).

Page 26, line 17: "Feng et al. 2021, Paper in submission". If possible, please update the reference. Even a preprint is helpful.

Response: This paper has not been published in a journal. It was posted at bioRxiv in 2021. We added the bioRxiv information of this paper in the reference list of the revised Manuscript.

Reviewer #2:

Zheng et al. generated whole-genome sequence data from 1,064 individuals of indigenous Tibetans. After applying strict quality filters to the data, 1,001 genomes were subsequently used for scanning selection signals. More than one third of 35 million variants identified from this data set (30 million SNVs and 4.8 million INDELS) have not been previously annotated in any other human populations. Thus, this dataset provides an unprecedented opportunity to uncover novel genes associated with high- altitude adaptation. With the rigorous tests of selection, this study was able to provide a high-confident list of 4,320 variants across 192 genes (including 158 genes newly identified), which are enriched with the functions related to cardiopulmonary system, oxygen sensing, development, and reproductive viability. This is the largest whole- genome sequence data from Tibetans, as far as I am aware of, and the discoveries made in this study, in addition to the data produced, will be critical to improve our understanding on the long-standing mystery on high-altitude adaptation. Overall, I am convinced with the results presented in this manuscript and only have two major comments below.

The mean depth reported in this study is 11.8x, which may not be sufficient to confidently call SNVs with rare alleles. Particularly, if those alleles are uniquely present in the Tibetans, it can be very challenging to prove that they are not false positives. This could be less problem if all Tibetan individuals are pooled together for genotype calling. However, this does not seem to be the case in this study as the calling was done for each individual separately (see Page 22, Lines 25-26). How would the authors be confident with rare variant calling given that the majority of newly identified alleles are rare in the population (Fig. 1c)?

Response: We understand and agree with the reviewer’s point on rare variants. In our study, the Best Practices Workflows of variant discovery by standard GATK was used. We called variants per-sample using the HaplotypeCaller module of GATK, and then we performed joint genotyping and applied VQSR filtering to produce the final multi- sample call-set with the desired balance of precision and sensitivity. As the reviewer mentioned, we believe joint calling and VQSR would reduce potential false positives for rare variants. For clarification, we rephrased this point in the Methods section of the revised manuscript (Page 20, Line 5-9).

Given that the authors show the polygenic nature of high-altitude adaptation, it would be very interesting to see if there are any traits (e.g. blood hemoglobin level), based on UK BioBank or BioBank Japan, that have signatures of polygenic selection in this population. There are useful methods available for this analysis (https://github.com/FerRacimo/PolyGraph; Berg and Coop 2014 PLoS Genet).

Response: Following the reviewer’s suggestion, we employed the Poly-Graph [R1] tool to identify signatures of polygenic selection in Tibetans. We retrieved a set of SNVs (3,643 SNPs) associated with blood hemoglobin level (HGB) using summary statistics from the large-scale GWAS meta-analysis of UK BioBank. To obtain a list of independent SNPs, we removed those SNVs in LD (r2 > 0.2 in 1KGP CEU). Finally, we obtained 2,031 independent HGB-associated SNPs. At the same time, we generated a set of 799,055 independent SNVs of Tibetans as the control panel. The PolyGraph result showed that Tibetans have a clear signature of polygenic selection on the HGB level (padjust = 1.38e-20). Considering that EPAS1 has a significant contribution to the Tibetan HGB as demonstrated in our previous study [R2], we also conducted the analysis by excluding the EPAS1 SNVs (gene body plus the upstream and downstream flanking 5 Kb regions). Consistently, the signature of polygenic selection remained unchanged (padjust = 1.13e-12). Bonferroni-correction was applied to the adjustment of multiple tests (see the added Figure S9 in the revised manuscript). In the revised manuscript, we added a paragraph to describe the result (Page 15, Line 2-9).

Minor comments:

1. I am not sure if "relocate" is a right word to represent what was done in this study as my understanding is that this study is refining the selection signatures in Tibetans with the largest set of whole-genome sequence data.

Response: In this study, we identified a new set of genes under Darwinian positive selection in Tibetans using large-scale WGS data, and among the 192 genes, 158 of them (82.3%) are newly identified genes, which greatly change the picture of genome- wide signals of selection in Tibetans. Therefore, we used the word “Relocate” to describe our findings.

2. Repeatability -> reproducibility?

Response: Yes, “reproducibility” is a better phrase, and we changed it accordingly in the revised manuscript (Page 2, Line 4).

3. Page 19, Lines 5-8: I don't think 1KGP3 data are biased towards European ancestry given that these are sequence data from global populations including African, Asian, and American ancestry as well. Any bias detected in this study is due to a lack of Tibetan ancestry in the panel for imputation.

Response: We agree with the reviewer, and we rephrased this sentence as “The commonly used human population reference panels (1KGP3) may bring bias for imputing genotypes of underrepresented populations (such as Tibetans), leading to distortion of population-specific genetic architecture.”

(Page 16, Line 14-17) 4. Page 24, Line 22: IMPUT2 -> IMPUTE2

Response: We corrected it accordingly.

5. In Fig. S4, where can the results on SVs be found?

Response: We have provided information of all the TESVs in Table S14. For the results of the genome-wide SVs, we provided an additional data in the revised manuscript (Additional file 1: Table S16).

Reviewer #3:

Zheng et al. whole genome sequenced 1001 Tibetan individuals. They detect selection using the CMS score, and do enrichment analysis to gain insight into what pathways are involved in this adaptation. I was excited to read this manuscript, since this is the largest set of whole genomes from a Tibetan sample, and Tibetans are an interesting population to study natural selection and demographic history. The reason is that Tibetans are adapted to high altitude, and several studies have suggested that these are genetic adaptations (not acclimatization), and studies have identified some of the loci underlying this adaptation. In addition, archaic introgression from a Denisovan-like population likely introduced (via introgression) the beneficial variation (at the EPAS1 gene) which is the strongest signal of selection in Tibetans.

This paper was easy to read, but the analyses and findings presented are underwhelming. The authors have access to the largest sample of Tibetans and focused their study on summarizing and cataloging variation, and it reads like a genome paper. The current findings presented do not provide a significant advance over previously published studies. In my opinion, the authors could have done more to make their study more interesting by studying the landscape of archaic introgression in this sample, and whether archaic variants influenced other genes involved in their high- altitude adaptation (in addition to EPAS1).

Response: We appreciate the reviewer’s approval of our study. According to the reviewer’s suggestion, we conducted introgression analysis, and searched the introgressed regions of archaic hominins in the Tibetan genomes using two commonly used tools: ArchaicSeeker2.0 [R3] and SPrime [R4].

The results show that Tibetans have similar genome-wide introgression levels (0.06%- 0.18% from Denisovan and 0.34%-0.79% from Neanderthal) with other non-African populations [5]. In the Tibetan genomes, we identified 599 (by ArchaicSeeker 2.0) and 670 (by SPrime) introgression regions, respectively. However, only 25 regions are shared between the sets generated by the two tools, which we take as the high-confidence introgression regions. Among the 25 high-confident introgression regions, only 5 regions harbor genes with signals of positive selection, and all of them have been reported in previous studies [R5], including EPAS1, EGLN1, DISC1, PRKCE and FANCA (the added Figure S6 in the revised manuscript; Additional file 1: Table S15). However, it should be noted that the detection of introgression region heavily depends on the haplotype information, which requires deep sequencing data (>30x) for high- quality phasing. Considering the relatively low sequencing depth (~11.8x) of our data, we could not confidently identify the genome-wide introgression regions, which was why introgression analysis was not included in the manuscript. Indeed, as pointed out by the reviewer, introgression analysis in Tibetans is very important and challenging, which call for future deep-enough sequencing (both short-read and long-read sequencing) data of Tibetans. In the revised manuscript, we added a paragraph to describe the results of introgression analyses (Page 10, bottom paragraph)

Some minor points:

Page 5, Line 8: "The genome-wide heterozygosity of Tibetans is 1.41" What are the units of 1.41?

Response: The genome-wide heterozygosity is the number of heterozygous sites in an individual divided by the number of the homozygous sites. It is a ratio. We rephrased the sentence in the revised manuscript (Page 5, Line 8).

Page 6, Line 3: "The known and novel variants are shown in red and blue respectively." The legend in the figure says the red are novel and blue are known.... Which does not make sense since we expect the novel ones to be low frequency.

Response: This was a labeling error in Panel-C of Figure 1. We got it corrected in the revised manuscript.

Page 8, Lines 11-16: Why focus on these three SNPs? I assume that if FST is high, these SNPs have been identified in previous studies? Are these SNPs in any interesting genes that haven't previously been identified?

Response: In common practice, when calling SNPs in populations without known natural selection, if a SNP shows a significant HWE deviation (p < 1e-6), it means that the calling of this SNP is not reliable since it deviates from Hardy-Weinberg equilibrium (HWE) (mostly due to sequencing or genotyping errors). In the Tibetan genomes, we identified 207 such SNPs (p < 1e-6). Because Tibetans have been under strong natural selection, this can also cause HWE deviation of SNPs besides of genotyping errors, especially for those SNPs under strong selection. To rule out that these SNPs are in poor-quality, we selected three high Fst SNPs and conducted Sanger-sequencing, and we confirmed that their genotype callings are highly confident. These three SNPs were only used to show the high calling quality of our SNP set.

References

R1. Racimo F, Berg JJ, Pickrell JK: Detecting Polygenic Adaptation in Admixture Graphs. Genetics 2018, 208:1565-1584. https://doi.org/10.1534/genetics.117.300489

R2. Peng Y, Cui C, He Y, Ouzhuluobu, Zhang H, Yang D, Zhang Q, Bianbazhuoma, Yang L, He Y, et al. 2017. Down-Regulation of EPAS1 Transcription and Genetic Adaptation of Tibetans to High-Altitude Hypoxia. Mol Biol Evol 34:818-830.

R3. Browning SR, Browning BL, Zhou Y, Tucci S, Akey JM: Analysis of Human Sequence Data Reveals Two Pulses of Archaic Denisovan Admixture. Cell 2018, 173:53-61 e59. https://doi.org/10.1016/j.cell.2018.02.031

R4. Zhou Y, Browning SR: Protocol for detecting introgressed archaic variants with SPrime. STAR Protoc 2021, 2:100550. https://doi.org/10.1016/j.xpro.2021.100550

R5. Zhang R, Yuan K, Xu S: Detecting archaic introgression and modeling multiple- wave admixture with ArchaicSeeker 2.0. STAR Protoc 2022, 3:101314. <https://doi.org/10.1016/j.xpro.2022.101314>

**2^nd^ round**

**Reviewer 1**

The current revision has addressed all the issues raised by me and the other reviewers with clarification, rewriting, and additional analysis. Notably, the authors added the polygenic adaptation analysis of blood hemoglobin level and the identification of introgressed regions of archaic hominins. I praised the authors' effort in adding these analyses. Honestly, the release of the high-quality large sample of whole-genome sequencing data for a minority population is by itself very exciting. It provides a reference for future evolutionary and clinical studies in this population (such as imputation). The long list of candidate variants and genes under selection in Tibetans significantly improves our understanding of genetic adaptation to the high altitude. This dataset offers many future research opportunities in this unique population, such as demographic history, archaic admixture, and disease genetics. The manuscript is of publication quality.

**Reviewer 2**

The authors provided sufficient responses to my comments. I do not see any other issues in the paper and am now happy to recommend acceptance of this paper.
